# SPADE: A Deep Learning Framework for Spatial Mapping and Quantitative Cell–Cell Interaction Inference

**DOI:** 10.1002/advs.76142

**Published:** 2026-06-18

**Authors:** Xinyi Li, Ning Zhang, Zijie Jin

**Affiliations:** ^1^ Department of Immunology School of Basic Medical Sciences Health Science Center Peking University Beijing China; ^2^ Health Science Center Peking University International Cancer Institute, Peking University Beijing China; ^3^ Translational Cancer Research Center Peking University First Hospital Beijing China; ^4^ Yunnan Baiyao Group Co., Ltd Kunming China; ^5^ School of Mathematics and Statistics Beijing Institute of Technology Beijing China

## Abstract

Spatial transcriptomics (ST) enables the study of tissue architecture by resolving gene expression in space, but current ST platforms are constrained by limited sequencing depth and indirect single‐cell identification. Existing deconvolution methods that integrate single‐cell RNA sequencing (scRNA‐seq) data with ST often overlook the biological principle that cells in communication with each other tend to be closer spatially. Here we introduce SPADE, a deep learning framework that aligns scRNA‐seq data to spatial locations by jointly modeling expression similarity between scRNA‐seq and ST data and concordance between the spot distance and cell–cell communication (CCC) patterns. SPADE also enables quantitative characterization of CCC across spots and regions. Evaluations on 55 simulated and real datasets show that SPADE achieves strong performance in recovering region‐specific cell‐type patterns and enhancing spatial gene expression profiles compared with existing methods. In the breast cancer datasets, SPADE demonstrates a unique advantage in identifying tumor‐infiltrating immune cells and tertiary lymphoid structures. In the colorectal cancer liver metastasis dataset, SPADE distinguishes tumor heterogeneity with region‐specific CCC events and describes the general CCC landscape in the tissue. Overall, SPADE highlights the key role of spatially constrained CCC in shaping tissue organization and enables biological interpretation of spatial transcriptomics data.

## Graphical abstract

SPADE integrates spatial transcriptomics with single‐cell RNA sequencing by using cell–cell communications (CCC) as a guide for spatial mapping. It improves cell‐type localization, enhances sparse gene‐expression signals, and reveals CCC programs at single‐spot resolution. Applications across well‐organized and complex tissues uncovered spatial distribution patterns, including immune infiltration in tertiary lymphoid structures, and heterogeneous tumor subregions with diverse CCC events.

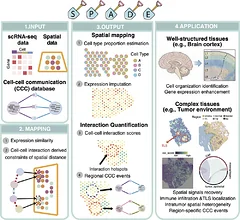

## 1 Introduction

Spatial transcriptomics (ST) is a revolutionary technology in biological research [1, 2], enabling simultaneous capture of transcript abundance and its location within tissue sections [3]. This capability is particularly valuable in oncology, supporting the analysis of tumor heterogeneity, mapping distinct cellular niches, and elucidating tumor microenvironment organization [4]. By identifying region‐specific transcript abundance signatures, ST also reveals spatially coordinated regulatory networks and intercellular interactions.

Although ST preserves spatial information, high‐resolution platforms often suffer from low transcript capture efficiency, while low‐resolution methods may capture transcripts from a mixture of cell types [5]. In contrast, scRNA‐seq offers high transcript capture efficiency at single‐cell resolution, but lacks contextual spatial information, limiting its application in studying tissue organization and cellular context. Integrating scRNA‐seq data with ST data has therefore become a widely adopted strategy to overcome the limitations of each individual technology. Computational methods [6, 7, 8, 9, 10, 11, 12, 13, 14, 15, 16, 17, 18, 19, 20], such as RCTD [18], cell2location [19], and Tangram [20], have been developed to deconvolute ST data using single‐cell reference datasets from similar or identical tissue samples (a “matched” dataset). However, current deconvolution methods face several limitations that diminish their utility: (1) Direct comparison between original and reconstructed expression profiles reveals that deconvolutions are highly sensitive to technical noise and that inclusion of uninformative genes frequently results in unstable cell mapping; (2) reliance on pre‐defined rules regarding cellular distribution patterns to guide deconvolutions may not be universally applicable, for example, the assumption that neighboring spots have similar cell type compositions often fails at tissue boundaries where cellular architecture exhibits abrupt rather than gradual transitions; (3) failure to leverage the biological principle that biologically interacting cells are more likely to be spatially co‐localized [21]. Without explicitly modeling cell–cell communications (CCCs), current methods may incorrectly assign transcriptionally similar but functionally unrelated cells to adjacent spatial locations, thereby confounding downstream analyses such as the inference of spatial signaling networks or the identification of microenvironmental niches. Recent methods like NODE [22], SIMVI [23], and scHolography [24] incorporate CCC measurements into spatial mapping, but they treat CCC as unknown variables, primarily due to the lack of a standardized and broadly accepted CCC quantification framework.

To address these limitations, we propose SPADE (SPAtial Deconvolution and CCC Evaluation), a computational framework for ST reconstruction and quantitative characterization of CCC across spots and regions by integrating scRNA‐seq data with ST data. Innovatively, SPADE introduces cell–cell interaction‐informed constraints of spatial distance to encourage cells with strong predicted communications to be mapped closer in physical space, thereby reconstructing a spatial landscape that is biologically meaningful and reflective of both molecular and intercellular interaction networks. Through cell mapping and gene imputation, SPADE outputs spatial profiles with increased read counts and single‐cell resolution, enabling the detection of more spatially informative genes and more accurate delineation of biologically significant spatial domains. Besides, SPADE also includes a module to quantify CCC intensity at single‐spot resolution directly, facilitating the accurate identification of region‐specific CCC events in tissue architecture. SPADE showed competitive or superior performance across simulated datasets with known ground truth, while exhibiting robust concordance with anatomical structures, canonical marker distributions, and reference‐method benchmarks in real datasets. It achieved robust single‐cell mapping across brain tissues with well‐defined laminar architecture and in tumor microenvironments with complex spatial organization, highlighting its adaptability across various tissues. In breast cancer, SPADE showed great potential to delineate the spatial organization of tumor‐infiltrating immune cells, identifying tertiary lymphoid structures (TLS) more precisely than other methods. In a colorectal cancer liver metastasis (CRCLM) dataset, the reconstructed spatial profiles successfully distinguished the spatial architectures of different tumor subtypes and specific CCC characteristics correlated with clinical outcomes. Moreover, SPADE is compatible with diverse spatial transcriptomic platforms, including high‐resolution ST datasets.

## 2 Results

### 2.1 Overview of SPADE Pipeline

SPADE takes as input a gene‐by‐spot expression matrix with spatial coordinates from a spatial transcriptomic dataset, and a gene‐by‐cell expression matrix with cell annotations from a matched scRNA‐seq dataset. The objective of SPADE is to align the matched scRNA‐seq data to the ST data by considering both gene expression similarity and the concordance between predicted cell–cell communication events and spatial proximity.

**FIGURE 1:**
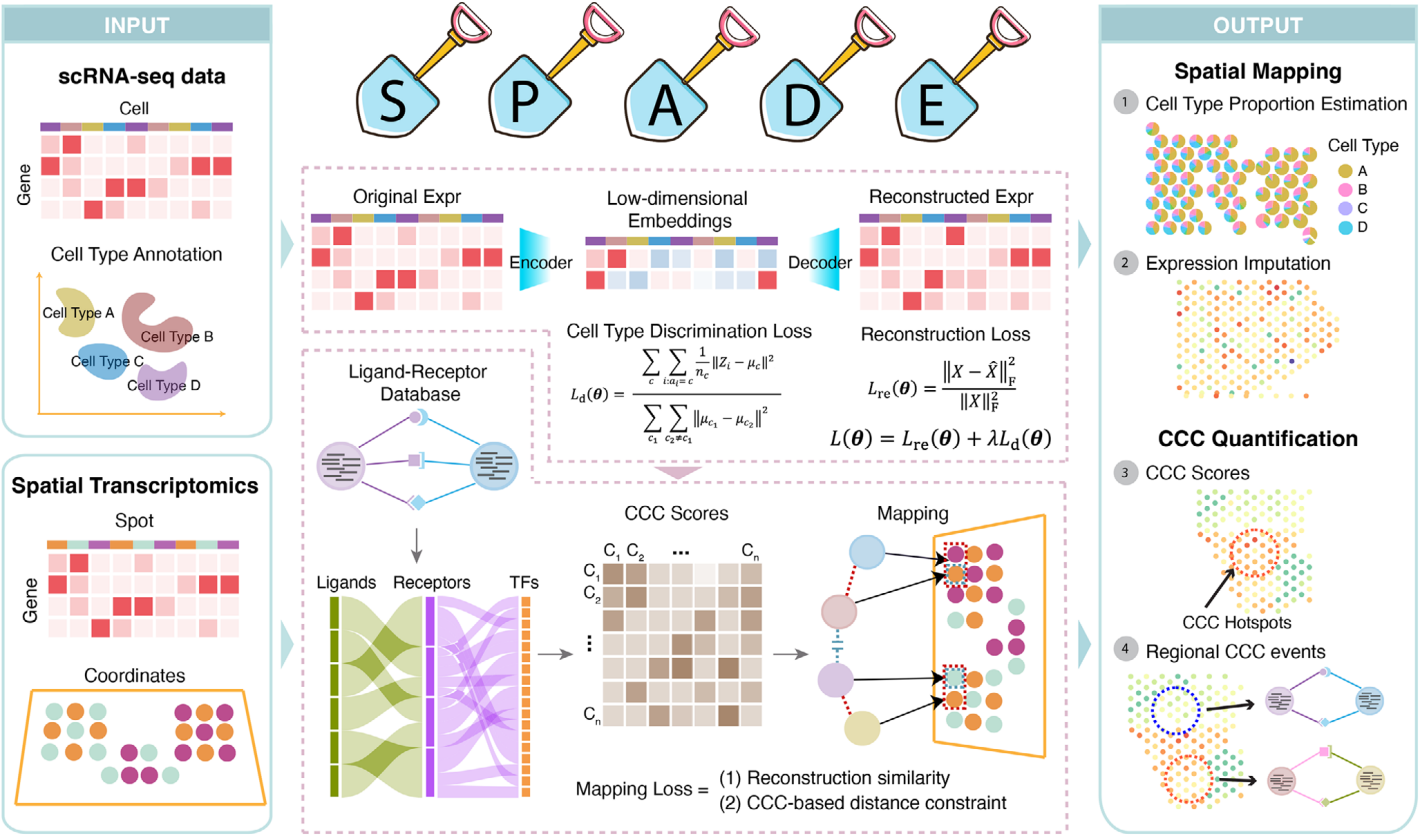
The SPADE pipeline. SPADE takes as input an ST dataset and a matched scRNA‐seq dataset with cell annotations (left). A deep‐learning module (upper center, Methods) then learns the low‐dimensional embedding of single cells and spots. The module consists of an autoencoder and a discriminator that extracts the expression features most relevant to cell types and reduces the expression noise for better alignment. Subsequently, SPADE calculates the CCC scores between two single cells using the ligand–receptor database and aligns cells to spatial locations according to the similarity of the embedding and the concordance of CCC scores and distances (lower center, Methods). SPADE outputs (1) the estimated cell type proportion in each spot, (2) the enhanced gene expression profile, (3) the CCC hotspot regions, and (4) regional CCC events (right).

The SPADE workflow is organized into three steps (Figure 1, Methods). In the first step, SPADE learns low‐dimensional embeddings of the gene expression profile using a deep‐learning model. The purpose of dimension reduction is to extract key features distinguishing different cell types while reducing computational burden. The deep‐learning model consists of an autoencoder and a supervised discriminator. Both the encoder and decoder of the autoencoder have two fully connected layers, with a 128‐dimensional bottleneck layer in between. The autoencoder captures compact representations of transcript abundance while preserving key structural patterns. The bottleneck layer is connected to a discriminator that maximizes the separation between different cell types while promoting tight clustering within each cell type. The model is optimized using a combined loss function consisting of a reconstruction loss from the autoencoder and a discrimination loss from the discriminator. The resulting low‐dimensional embeddings in the latent space are subsequently used in downstream modules.

In the second step, SPADE calculates a cell–cell communication score between each possible pair of single cells. The CCC score for a cell pair is essentially the sum of ligand–receptor (L–R) gene pairs’ activities across the two cells. However, not all L–R pairs contribute equally to the CCC score. SPADE assigns weights to L–R pairs according to their expression specificity in the dataset, under the assumption that L–R pairs enriched in particular cell groups often indicate more functional intercellular interactions and are more informative for their spatial constraints, whereas those with uniform expression across all cells are less likely to be functionally relevant and spatially localized. To mitigate the potential for false positives from chance co‐expression of ligand and receptor genes, SPADE further calculates the correlation between the expression of ligand/receptor genes and the expression of genes known to be regulated by the L–R pair, and then multiplies the initial interaction scores by a function of the correlation coefficient (Methods). The final CCC score for each cell pair is obtained by summing the adjusted interaction scores from all L–R pairs.

In the third step, SPADE performs the alignment of scRNA‐seq data to the ST data, ensuring that the reconstructed ST data closely resembles the original data, and the single cells with strong interactions are positioned closer in spatial location. Technically, SPADE rewards spatial alignments that achieve high cosine similarity between the expression embeddings of the original ST data and the reconstructed ST data, as well as a high concordance between scaled spatial distances and reciprocal CCC scores. The alignment is a probabilistic mapping matrix, where the elements represent the mapping probability of a single cell to one spot. Using the alignment matrix, SPADE estimates the cell‐type proportion at each spot by aggregating the mapping probabilities of cells belonging to the same cell type. The reconstructed gene expression for the ST data is computed by multiplying the single‐cell expression matrix with the mapping probability matrix, followed by normalization to ensure uniform total gene counts across all spots. SPADE estimates the CCC activity at each spot by calculating the distance‐weighted sum of CCC scores between the target spot and all other spots, along with the statistical significance of each CCC score. SPADE also reports the CCC events that are specific to given spatial regions.

### 2.2 SPADE Accurately Maps Cells in a Simulation Study

**FIGURE 2:**
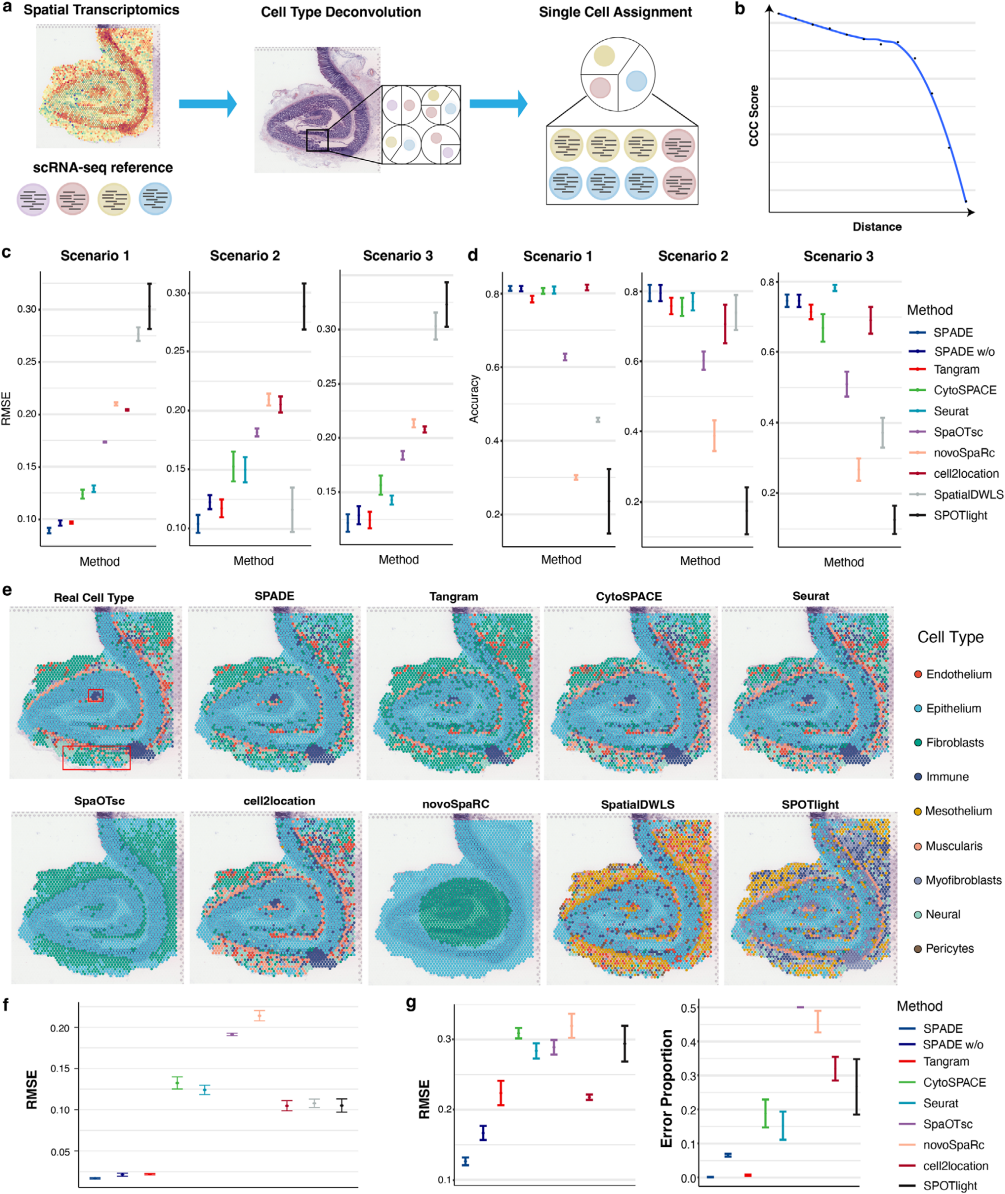
The simulation study. (a) The design of the simulation study. A spatial transcriptomic dataset and matched scRNA‐seq data were used to estimate the cell type distribution. Single cells were then assigned to spots according to the cell type proportion. Gene expression values of each spot were calculated by summing the normalized gene expression values of assigned single cells. (b) The estimated CCC intensity score between two spots with a given distance. (c) The RMSE of estimated cell type proportion and true cell type proportion. The error bar indicates the ± 1 standard deviation of the mean RMSE. (d) The accuracy of dominant cell detection. The error bar indicates the ± 1 standard deviation of the mean accuracy. (e) The estimated dominant cell type of each ST spot. (f) RMSE of estimated cell type proportion and true cell type proportion (Left) in the first additional simulation. (g) RMSE of estimated cell type proportion and true cell type proportion (Left). Proportion of cell types C1 and C2 that aligned to the incorrect region (Right). Error bar indicates the ± 1 standard deviation of the mean RMSE and error proportion. The method “SPADE w/o” represents SPADE with the CCC module turned off.

To evaluate the accuracy of SPADE in aligning single cells to spatial locations, we conducted a simulation study on a set of pseudo‐spatial data generated from real scRNA‐seq and ST data under various conditions, resulting in a collection of 40 simulated datasets (Figure 2a). To generate simulated datasets, we used a spatial transcriptomic dataset from intestinal tissue [25] as the expression template and a matched scRNA‐seq dataset [22] to generate a simulated “ground‐truth” for both cell‐type distribution and spatial gene expression, preserving the intrinsic cell context information observed in the real data (Methods). Specifically, we first performed cell‐type deconvolution using RCTD to obtain the estimated cell‐type proportion in each spot. Single cells were then randomly assigned to the spots according to these proportions by sampling from a multinomial distribution. The cell‐type composition and the aggregated gene expression of these assigned single cells were considered as ground truth. A gene expression count matrix was subsequently generated by sampling from a zero‐inflated negative binomial distribution, with mean values derived from the baseline expression and zero‐inflation/over‐dispersion parameters estimated from the real dataset. Random dropout events and spot swapping events were added for mimicking technical noise. To evaluate the performance of SPADE, we designed three scenarios to reflect varying degrees of mismatch between the scRNA‐seq and ST datasets (Methods). Scenario 1 represented a perfect match of cell types between scRNA‐seq data and ST data. In scenario 2, one cell type in the ST dataset was excluded from the single‐cell dataset. In scenario 3, the labels for two cell types were intentionally switched, simulating a user providing an incorrect cell‐type annotation in the scRNA‐seq dataset.

We first sought to demonstrate that the simulated datasets preserved the spatial context of the assigned cells by calculating CCC scores between spots. As expected, CCC scores dropped dramatically when the distance between two spots exceeded a threshold, confirming the relationship between CCC activity and spatial proximity (Figure 2b). Across the simulated datasets, the cell‐type compositions reported by SPADE were closest to the ground truth, followed by Tangram, Seurat, and CytoSPACE (Figure 2c, Figure S1a, and Table S1). SPADE had the best overall performance in determining the dominant cell type and in determining the two most abundant cell types in each spot (Figure 2d, Figure S2a, and Table S2). For example, epithelial cells are expected on the mucosal surface, and SPADE successfully predicted the highest number of epithelial cells in this region, whereas other algorithms predicted a higher proportion of other cell types (Figure 2e). Similarly, SPADE effectively detected immune cells, which several alternative methods failed to identify (Figure 2e, red box). Cell‐type proportions predicted by SPADE in this immune‐infiltrated region were also most consistent with the ground truth. Among the algorithms capable of reconstructing spatial gene expression, SPADE had the overall highest correlations between reconstructed gene expression and original gene expression (Figure S2b). In scenarios 2 and 3, the performance of all nine methods was reduced, with increased variance in all metrics, reflecting the increased task difficulty under imperfect reference conditions. Despite this, SPADE maintained the overall best performance and lowest variability across simulated datasets, demonstrating its robustness under imperfect conditions (Table S3).

We observed a consistent increase in root mean square error (RMSE) when the CCC module of SPADE was removed, whereas the detection of dominant cell types and the correlation between reconstructed and original gene expression remained largely stable (Figure 2c,d,f,g; Figure S1). This result suggests that the effect of the CCC module is to improve cell‐type proportion estimation and spatial co‐localization of interacting cell populations, rather than globally reshape the dominant cell‐type assignments or reconstructed expression profiles. To further investigate the effect of CCC events in aligning single cells to spatial locations and to determine whether SPADE captures CCC information, we designed two additional simulation studies. The first simulation is that we benchmarked SPADE against competing methods on 10 simulated datasets generated using scMultiSim with explicit CCC events [26]. SPADE achieved the lowest RMSE (ranging from 0.015 to 0.018) and highest Pearson's correlation in these datasets, outperforming other competing methods (Figure 2f; Figure S1b). SPADE without the CCC module (SPADE w/o) ranked second, and the performance gap between SPADE and SPADE w/o demonstrates that incorporating CCC information provides additional benefit beyond expression similarity alone. The second simulation is that we generated datasets containing only three cell types (A, B, and C), with cell types A and B located at the upper and lower regions of the spatial domain, respectively. We split the C cells into equal groups called C1 and C2, and then manually introduced CCC events such that C1 interacted with cell type A and C2 with cell type B (Methods). SPADE was the top performer in both cell type proportion estimation and C1/C2 alignment, demonstrating the contribution of CCC events in the alignment (Figure 2g; Figure S1c). Overall, this simulation demonstrated the accuracy and robustness of SPADE in mapping single cells to spatial locations, as well as the effectiveness of integrating CCC information into spatial alignment models.

### 2.3 SPADE Enables the Localization of Cell Types in Mouse Cerebral Cortex and Human Dorsolateral Prefrontal Cortex (DLPFC)

**FIGURE 3:**
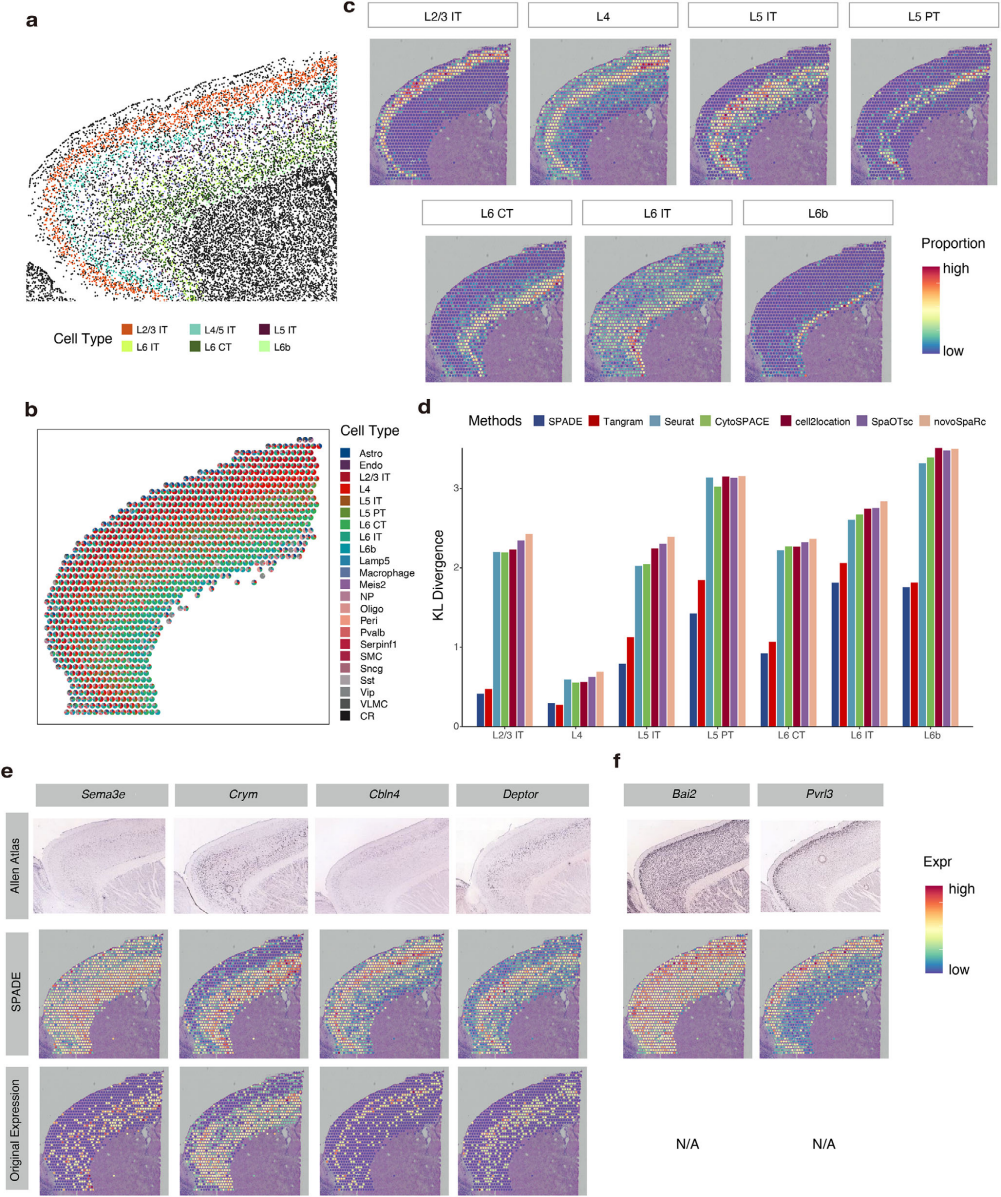
SPADE alignment in mouse cerebral cortex. (a) Regions containing different cortical layers in the anterior sagittal sections of the mouse brain. (b) Cell type composition of each spot estimated by SPADE. (c) The distribution of cell types known to be localized to certain cortical layers. (d) K–L divergence of estimated cell type proportions from given methods and RCTD on the regions of cortical layers. (e) Gene expression enhancement from SPADE. Top row: reconstructed gene expression; middle row: Original gene expression values; bottom row: in situ hybridization intensity values for the indicated genes from the Allen Brain Reference Atlas. (f) Predicted expression of two genes that were not detected in the original dataset.

We next applied SPADE to decompose cell types in real spatial transcriptomic datasets. We chose an anterior sagittal section of mouse cerebral cortex analyzed with the 10× Visium protocol. The dataset consisted of 1062 spots and 31 053 genes, with a median 6015 genes detected per spot. We used a scRNA‐seq reference dataset from an adult mouse cortical region that consisted of 14 249 cells with a total of 34 617 detected genes [27]. The cerebral cortex exhibits a highly structured, six‐layered architecture, with each layer defined by a distinct cellular composition (Figure 3a). Such a well‐organized structure allows for straightforward visualization and evaluation of alignment. The SPADE alignment yielded a significant enrichment of neuronal subtypes that are known to be present in the corresponding cortical layers (Figure 3b,c). For example, the L2/3 IT cell type is known to be enriched in the outer cortical layer [28], which was reproduced by the SPADE assignment, along with the enrichment of L6b cells [29] within the deepest layer (Figure 3c).

We then compared the performance of SPADE with other methods. Although the general cellular identity of each cortical layer is well established, an absolute ground truth defining the precise cell‐type proportions within these layers remains unavailable. We used the deconvolution result from RCTD as a reference for evaluation (Figure S3a). SPADE achieved the highest consistency with RCTD (Figure 3d; Figure S4a), with an average Kullback–Leibler (K–L) divergence value of 1.54. The remaining five methods showed much larger discrepancies among all cell types. NovoSpaRc failed to recover the laminar structure of the cortex, while SpaOTsc and cell2location partially captured spatial features but produced low mapping probabilities. Seurat introduced substantial noise for certain cell types (e.g., L6 CT and L6b) and lost other cell types completely. CytoSPACE exhibited reduced noise but resulted in a fragmented and discontinuous spatial distribution (Figure S3b and Table S4). Overall, SPADE exhibited the highest concordance with RCTD among these methods in spatial alignment across diverse cell populations.

Another function of SPADE is to impute the expression of genes that may have incomplete or noisy measurements in the original data. Using the mouse cortex datasets, SPADE imputed 16 949 additional highly variable genes (increasing from 16 013 to 32 962), with the average gene count per spot increasing from 5682 to 16 452. The reconstructed spatial transcriptomes retained the layer‐specific distribution of marker genes selected from the top differentially expressed genes across cortical layers in the Allen Brain Atlas [30] (Figure S5). Using RNA in situ hybridization data from the Allen Brain Atlas as ground truth [24], SPADE corrected discontinuous or ambiguous spatial gene expression patterns observed in the original spatial transcriptomics data (Figure 3e). For example, the L4 neuron marker gene *Cbln4* exhibited higher expression specifically within cortical layer 4 regions after SPADE reconstruction, which was consistent with the Atlas, whereas its spatial pattern was poorly defined in the original profile. SPADE also enhanced the signal of the L5‐specific marker gene *Deptor*, enabling a clearer and more continuous delineation of the L5 cortical layer compared to the original data.

SPADE predicted the expression of Spatially Variable Genes (SVGs) that were not detected in the original data (Figure 3f). For example, SPADE accurately reconstructed the spatial expression patterns of *Bai2* and *Pvrl3*, recovering their distribution in the outer cortical layers, consistent with the ground truth as defined by the Allen Brain Atlas. Using the same dataset, we tested existing deconvolution methods that also support gene imputation and found that these methods either reconstructed high expression of marker genes in incorrect cortical layers or failed to enhance relative gene expression in the expected regions (Figure S6).

We applied SPADE to another ST dataset from human dorsolateral prefrontal cortex (DLPFC) with a single‐nucleus RNA‐seq dataset created from three donors as reference [31] (Figure S7a). The DLPFC exhibits a six‐layered cortical organization, comprising layers I to VI and a white matter region, each with a distinct cellular composition. To define the ground truth for spatial mapping, we used the most abundant cell types for each cortical layer reported by the snRNA‐seq publication [25]. SPADE correctly aligned single cells to their corresponding known spatial locations, achieving the highest precision in cell‐type estimation and high Pearson's correlation with the result from RCTD (Figures S7b,c,S4b). Further, SPADE effectively recovered undetected gene expression profiles while simultaneously amplifying localized spatial patterns that were previously obscured in the original data. (Figure S7d,e).

### 2.4 SPADE Deciphers the Tumor Microenvironment and Immune Infiltration in Breast Cancer

**FIGURE 4:**
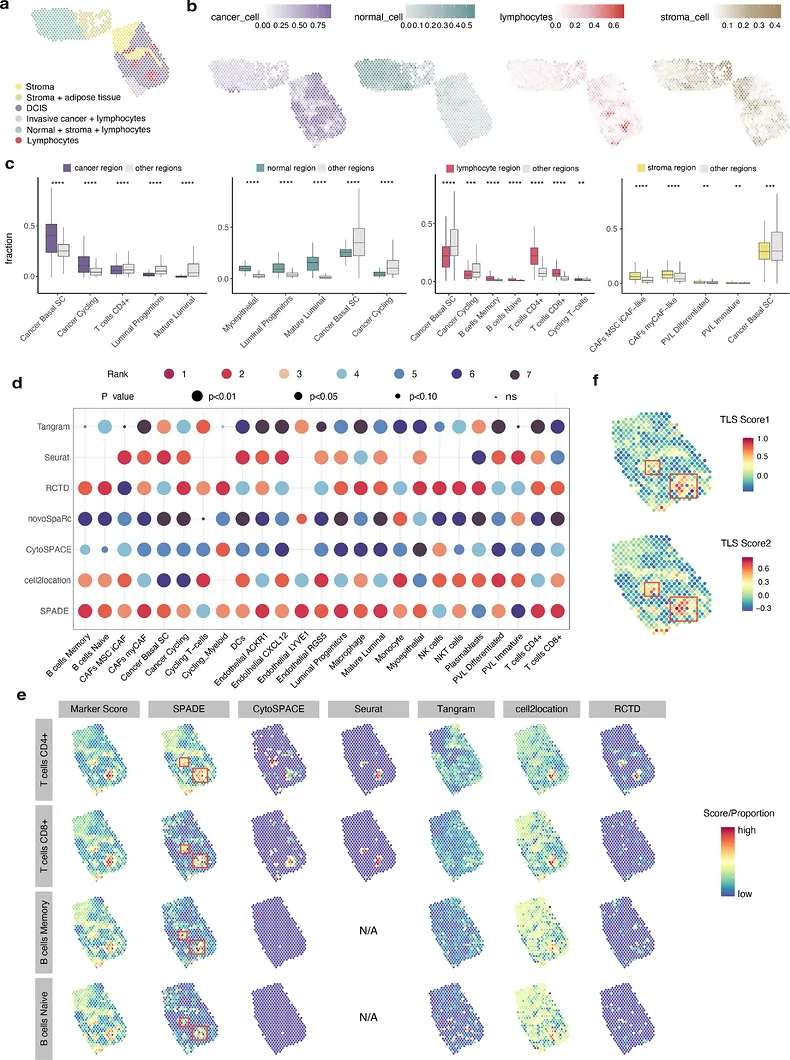
SPADE alignment of a TNBC dataset. (a) SPADE‐annotated spatial regions of TNBC data. (b) SPADE estimations of cell‐type proportions of four major cell types at each spot. (c) Comparisons of regional abundance among specific cell types in cancer, normal, lymphocyte, and stroma regions versus other regions. (d) Significance (point size) and rank (colors) of correlations between inferred cell‐type proportions and corresponding cell‐type‐specific marker genes across spatial locations for each algorithm. *p*‐values calculated through a two‐sided *t*‐test for the significance of Spearman's correlation. (e) Marker gene scores and cell type proportions for four cell types. (f) Gene signature score of 50 TLS genes (top) and 12 TLS chemokine signatures (bottom). Red boxes indicate potential TLS regions.

To evaluate the performance of SPADE in histologically complex tissues, we applied SPADE to a published triple‐negative breast cancer (TNBC) spatial transcriptomic dataset profiling 19 237 genes across 822 spots [32]. Based on the provided pathological annotations, the tissue section was stratified into six distinct histological domains: normal ductal, normal stromal, normal adipose, lymphocyte aggregates, ductal carcinoma in situ (DCIS), and invasive cancer (Figure 4a). Paired scRNA‐seq data from the same publication [32] covered 29 733 genes and 7986 cells. Cells were clustered into 25 subtypes, including two breast tumor subtypes and three normal mammary cell subtypes [32].

SPADE accurately captured the spatial distribution of four known major cell types in TNBC (Figure 4b). It correctly mapped normal breast cells to normal tissue areas and identified a significantly higher proportion of malignant cells within tumor regions than in non‐tumor regions. Immune cells, including B and T cells, were predominantly enriched in lymphoid‐like areas, while cancer‐associated fibroblasts (CAFs) were localized at stromal regions (Figure 4c). These mapping results highlight the reliability of cell‐type proportions derived by SPADE.

While most algorithms produced broadly similar cellular distributions, some spatial misalignments remained in several methods. For example, SpaOTsc misassigned normal cells to tumor regions, whereas novoSpaRc completely failed to preserve spatial domain features (Figure S8). In addition, the cell‐type proportions inferred by SPADE exhibited the strongest correlation with the expression of cell‐type marker genes, with Seurat ranking second and RCTD third (Figure 4d; Figure S9). However, Seurat excluded biologically relevant populations, such as the B cell, T cell, and NK cell subtypes, which were clearly localized by SPADE at tumor‐infiltrating lymphocyte regions. Further, Tangram and CytoSPACE incorrectly mapped normal breast cells to tumor regions, while cell2location distributed cell types nearly uniformly across spots (Figure S10a). Importantly, SPADE revealed that the invasive tumor area had higher immune cell infiltration than the DCIS region, indicating a strong immune response to tumor invasion (Figure S10b).

SPADE accurately resolved the spatial organization of diverse cell types, particularly regarding the immune cells within the tumor microenvironment. Using cell‐type–specific marker gene scores as ground truth, SPADE successfully captured the clustering of distinct B‐cell and T‐cell subtypes within lymphoid regions, particularly two spheroid‐like aggregations in the DCIS regions that suggest TLS formation [33] (red boxes, Figure 4e). These putative TLS regions were corroborated by the upregulation of 50 TLS‐associated genes and 12 chemokine signatures [34, 35] (Figure 4f). In contrast, the other methods partially or completely failed to capture the spatial distribution of certain cell subtypes or features, including the TLS (Figure 4e).

Using the paired scRNA‐seq dataset, SPADE reconstructed an enhanced spatial transcriptomic profile of TNBC, expanding gene coverage and significantly elevating the per‐gene transcript counts. SVGs identified from the reconstructed data exhibited higher spatial autocorrelation than the same set of genes in the original data (Figure S11a). 8587 genes were recognized as SVGs in both the original and reconstructed expression profiles, including *CD99* [36], a gene involved in immune cell migration and activation, and *S100A4*, which promotes pro‐tumor macrophage polarization [37]. SPADE effectively enhanced these gene expression levels and resolved their spatial patterns in the reconstructed data (Figure S11b). Further, SPADE identified 425 SVGs that were not detected in the original data, including integrin‐related gene ITGA4 [38] and the gene encoding T‐cell activation molecule *SKAP1*, both of which were enriched in lymphoid regions and regulate immune cell adhesion and migration [39]. Another newly identified SVG, *UQCRHL*, was highly expressed in tumor regions and functions to promote tumor progression through mitochondrial regulation [40] (Figure S12a). SPADE also identified 460 novel SVGs from imputed genes, such as ATP synthase subunits *ATP5E* and *ATP5L*, which were upregulated in tumor regions (particularly in DCIS), indicating enhanced metabolic activity of malignant cells. The SVG *FYB*, which plays an important role in T‐cell receptor‐mediated integrin‐dependent adhesion and is highly expressed in lymphocytes [41], showed a consistent spatial distribution in SPADE predictions (Figure S12b). GO analysis of SVGs identified from original and reconstructed data revealed an 80% overlap in enriched pathways (Figure S12c). Notably, the reconstructed profiles revealed more spatially coherent enrichment patterns related to tumor proliferative/metabolic programs and immune infiltration within the TME (Figures S12d and S13). For example, SPADE reconstruction enhanced the spatial contrast of cell cycle activity, including CDK‐related signaling, by reducing background signals in normal tissue while increasing pathway enrichment in tumor regions. Similarly, the B‐cell‐mediated immunity pathway showed a diffuse pattern in the raw data, whereas SPADE reconstruction resolved its localized activation within TLS‐like regions (Figure S13c,d). Collectively, these results support that SPADE reconstruction effectively recovers spatially variable signals and enhances the biological interpretability of spatial transcriptomic data in the tumor microenvironment.

### 2.5 SPADE Resolves Tumor Heterogeneity and Microenvironmental Differences in Colorectal Cancer Liver Metastasis

**FIGURE 5:**
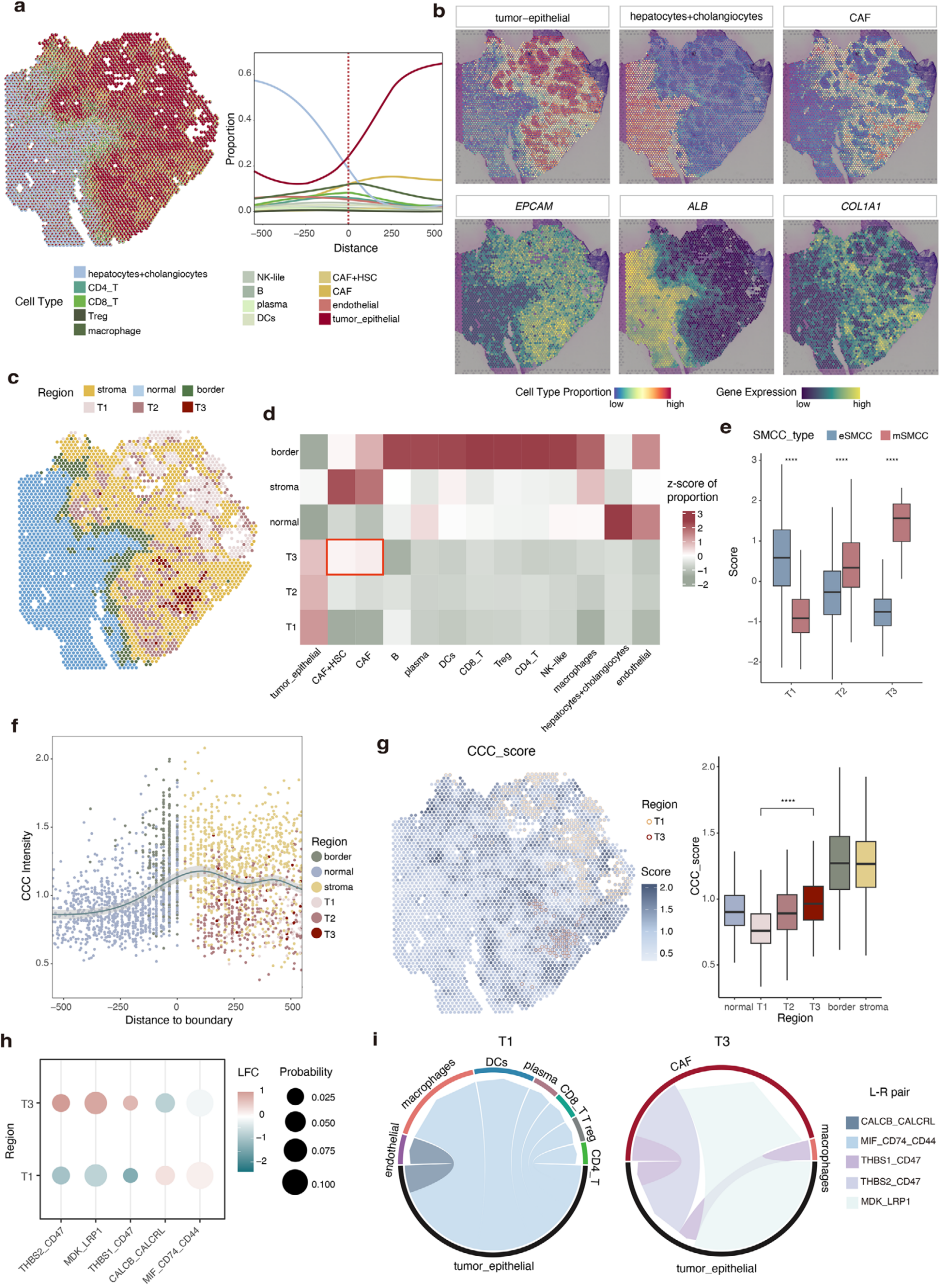
SPADE reveals regional cell–cell communication events in a CRCLM dataset. (a) Left: Pie plots of the inferred cell‐type composition at each spot by SPADE. Right: The predicted cell type distribution against the distance to the boundary. Negative distance represents the normal region on the left side of the boundary, while positive distance represents the tumor region on the right side of the boundary. (b) Top row: the proportion of region‐specific cell types inferred by SPADE. Bottom row: Expression levels of corresponding cell‐type‐specific marker genes. (c) ST data was clustered, and each cluster was annotated based on histological features (Methods). (d) The inferred proportion of cell types in each ST cluster. (e) eSMCC and mSMCC subtype scores across tumor clusters. (f) The CCC intensity against the distance to the boundary. Each dot represents a spot, and the color indicates its region annotation. The fitted curve stands for the mean CCC intensity of given distance. The shadow of the curve indicates the 95% confidence interval around the smooth. (g) Intercellular communication scores of each spot calculated by SPADE (left) and intercellular communication scores of each ST cluster (right). (h) Cell–cell communication results from CellChat. Colors represent log fold change (LFC) between the target region and other regions. (i) Chord diagram of ligand–receptor pairs enriched in T1 and T3 subregions between cell types. The colors represent different ligand–receptor pairs.

We applied SPADE to a 10× Visium CRCLM section capturing the invasion of colorectal cancer cells into the adjacent normal liver parenchyma [42]. By integrating matched scRNA‐seq data [43], SPADE reconstructed the spatial organization of cell types, which aligned with corresponding histological regions (Figure 5a left and Figure S14a). Specifically, hepatocytes and cholangiocytes were enriched in the albumin‐secreting normal liver regions, while tumor epithelial cells surrounded by CAFs clustered in regions with high expression of epithelial marker *EPCAM* and extracellular matrix (ECM) marker *COL1A1*, respectively (Figure 5b). Immune cells such as B cells, T cells, and NK cells colocalized at the border region between normal liver and tumor (Figure S14). To explore spatial characteristics along the tumor invasion gradient, we refined the tumor‐normal boundary based on histological features and calculated the shortest distance from each spot to the boundary (Figure S15a). Hepatocytes and malignant cells had mutually exclusive spatial distributions, and most of the immune cells were concentrated near the interface (Figure 5a right).

Systematic comparison of SVGs between the original and SPADE‐reconstructed profiles validated the capacity to effectively amplify spatial transcriptomic signals while preserving biological fidelity. SPADE recovered 6244 SVGs from the raw data and enhanced spatial autocorrelation of new SVGs (Figure S16a). Meanwhile, SPADE identified 724 genes as new SVGs that were not detected in the raw data, including *FCN2*, a key innate immune molecule synthesized primarily by the liver, whose expression was specifically enhanced in the hepatic regions after reconstruction (Figure S16b). Among the genes imputed by SPADE, 73 were identified as new SVGs. *APOC4‐APOC2*, which encode apolipoproteins, exhibited distinct expression in hepatic cells after reconstruction, consistent with known physiological functions of the liver. Similarly, the reconstructed signal of *CTGF*, which is mainly secreted by CAFs, was concentrated within the stroma, reflecting its known role in ECM remodeling (Figure S16c).

To detect spatial domains, we performed unsupervised clustering on the reconstructed spatial transcriptomic data using Seurat (Methods). Six clusters were identified, which we annotated based on histological features from the original study [42]: normal liver, tumor‐normal interface (border), tumor‐associated stroma, and three tumor subregions (T1, T2, and T3) (Figure 5c; Figure S15b,c, Methods). We then examined the expression patterns of the anatomical marker genes [42], revealing high *ALB* expression in the normal liver, *EPCAM* enrichment in the tumor region, and a stroma region characterized by abundant *COL1A1* levels (Figure S15c), which remained highly consistent between raw and reconstructed profiles, with a mean Pearson's correlation coefficient of 0.783 (Figure S17a). We further performed differential expression analysis for each cluster, and the resulting molecular signatures were highly concordant with their expected biological characteristics (Figure S17b and Table S5). Cell types were mapped appropriately within these regions, with tumor epithelial cells enriched in the tumor subregions, hepatocytes localized predominantly in the normal region, CAFs abundant in the stromal area, and diverse immune cell types accumulated at the border (Figure 5d).

We performed GO and KEGG enrichment analysis on the four major regions. The border region was enriched in genes and signaling pathways related to immune activation and lymphocyte development, suggesting active immune surveillance at the invasive front (Figure S18a). The normal region expressed genes involved in hepatic functions, including metabolic enzymes such as *CES2* and CYP4F3 [44] (Figures S18b,S19). ECM‐related pathways were upregulated in the stroma region (Figure S18c). Tumor areas showed high levels of intestinal epithelial markers and colorectal cancer stemness genes like *CD24* [45], along with activation of oncogenic pathways associated with cell cycle and DNA replication (Figures S18d,S19). Collectively, these outcomes confirm that spatial domains resolved from reconstructed profiles reflect both the histological architecture and the intrinsic functional features.

SPADE divided the tumor itself into three regions, revealing a spatial pattern underlying tumor heterogeneity. We evaluated the gene signatures associated with previously defined subtypes of senescent metastatic cancer cells (SMCCs) in colorectal cancer [39]. The epithelial‐like SMCC (eSMCC) phenotype [39] aligned with T1 tumor cells, whereas T3 tumor cells were dominated by a mesenchymal‐like SMCC (mSMCC) profile, which is related to recurrence and poor prognosis [39] (Figure 5e). Prior studies have shown that TGF‐β secreted by CAFs and M2 macrophages can induce the mSMCC state [39], consistent with that CAFs were prominently enriched within the T3 tumor subregion (Figure 5d,e; Figure S15d).

We benchmarked SPADE against other methods to evaluate its capability in resolving tumor heterogeneity. We clustered the reconstructed spatial transcriptomic data from each method using a unified pipeline, identifying tumor‐associated groups based on the paired histopathological image, followed by eSMCC and mSMCC subtype scoring (Figure S20). The clusters inferred by novoSpaRc showed no correspondence with the histopathological features, while Tangram and SpaOTsc failed to delineate distinct spatial distribution patterns of the two tumor subtypes. Although CytoSPACE partially reflected such patterns, it produced fragmented tumor subregions with poor spatial continuity and excessive subdivision. Seurat performed comparably to SPADE, providing external validation. Collectively, these findings indicate that SPADE accurately captures the spatially distinct patterns underlying tumor heterogeneity.

### 2.6 SPADE Captures CCC Events Related to Prognosis in Heterogeneous Tumors

The CCC scoring module of SPADE not only improves spatial mapping but also provides a standardized approach to quantify intercellular communications across the spots or tissue. We calculated CCC scores at single‐spot resolution to compare interaction intensities of spatial domains. The border and stroma regions showed the most active intercellular communications (Figure 5f,g). Interactions at border regions were primarily associated with cell adhesion, immune cell chemotaxis, and proliferation (Figure S15e and Table S6), suggesting that tumor invasion triggers a robust local immune response and stromal remodeling. We investigated the difference in CCC scores among tumor subregions, particularly between T1 and T3, which demonstrated the most significant pairwise difference (Figure 5g). To elucidate its molecular mechanisms, we identified ligand–receptor pairs significantly enriched in each subgroup (Figure 5h,i and Tables S7,S8). We found that *THBS1/2_CD47* and *MDK_LRP1* were upregulated and co‐expressed in T3, which is consistent with prior research showing that the ligand (MDK) and its receptor (LRP1) can activate CAFs, forming a pro‐tumorigenic microenvironment [46]. Prior research has shown that *THBS* binds to *CD47* on cancer cells, activating the “don't eat me” signal that facilitates immune evasion [47]. GO enrichment analysis for ligand and receptor genes that were highly activated in T3 yielded pathways related to tumor progression (Figure S21a). These results offer clues regarding possible mechanisms for recurrence of tumors with mSMCC phenotypes. In the T1 subtype, tumor epithelium exhibited extensive interactions with multiple cell types, prominently mediated by the MIF signal (Figure 5h; Figure S21b). A recent study using MIF‐knockout colorectal cancer mouse models demonstrated limited tumor growth and the reduction of both CD4^+^ and CD8^+^ regulatory T cells (Tregs) in MIF‐deficiency microenvironment [48], supporting the potential of anti‐MIF therapy, particularly for the eSMCC subtype. Thus, SPADE analysis of CCC revealed a fundamental difference in immune evasion strategy between the T1 and T3 tumor subregions, with potential clinical implications.

### 2.7 SPADE Is Compatible With High‐Resolution ST Data

In higher‐resolution ST protocols, the diameter of the spatial spots is smaller, often comparable to the diameter of individual cells. This makes it possible to annotate each spot with a single cell type rather than estimating cell type proportions [49]. To enable comparisons across different methods, SPADE reports both the proportion of cell types in each spot (cell‐type proportion) and the single cell type that has the highest proportion in each spot (dominant cell type).

**FIGURE 6:**
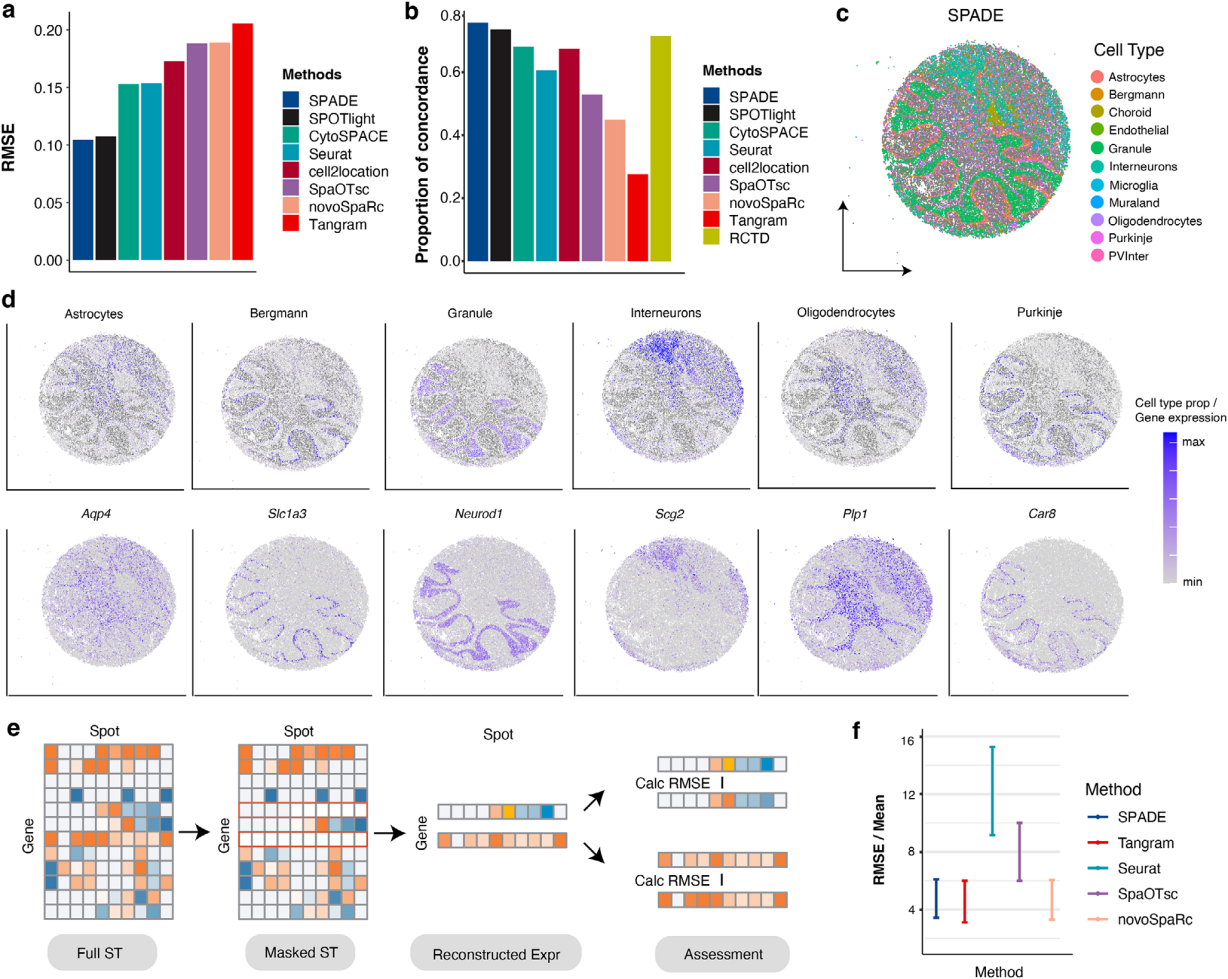
SPADE compatibility with high‐resolution ST data. (a) The RMSE of estimated spot type proportion and consensus spot type proportion from a Slide‐seq V2 mouse cerebellum dataset. (b) Proportion of concordance of estimated spot type (dominant cell type). The reference dominant cell type was obtained from the majority consensus across methods. (c) Spot type distribution annotated by SPADE. (d) The distribution of selected cell types and the expression of their marker genes. (e) Gene expression reconstruction benchmark design. We deleted 20 randomly selected genes from the ST dataset and assessed performance by calculating the RMSE between reconstructed and original gene expression data. (f) The RMSE divided by mean expression was used to measure performance. Error bar indicates the ± 1 standard deviation of the mean.

We applied SPADE and other currently available algorithms to a Slide‐seq V2 dataset collected from mouse cerebellum tissue, which has a near single‐cell resolution [50, 51]. This dataset contains over 30 000 spots with a median of just 196 genes detected per spot. The sparsity of high‐resolution ST datasets significantly hinders spot type annotation and downstream analysis, making enhancement of the data through integration of scRNA‐seq data critical. The cell type proportions estimated by SPADE had the highest concordance with those estimated by RCTD, whereas Tangram, which achieved the second‐best performance in the simulation benchmark, had greater deviations from RCTD estimates (Figure 6a). We used the majority consensus across methods as a practical reference to compare dominant cell‐type assignments, which represented a consensus‐based concordance comparison rather than a validation against an independent gold standard. SPADE showed the highest concordance with the majority consensus among all evaluated methods (Figure 6b). The spot annotations derived by SPADE were also consistent with the known spatial architecture of mouse cerebellum, including assignment of granule cells, Bergmann cells, and Purkinje cells (Figure 6c). RCTD, SPOTlight, CytoSPACE, and cell2location did not outperform SPADE, but achieved better annotations than other currently available methods (Figure S22). Tangram failed to detect interneurons, while Seurat detected the granule layer and Purkinje layer with higher levels of noise than SPADE. In addition, we examined whether the spatial distribution of each cell type predicted by methods was consistent with the expression patterns of their canonical marker genes in the original ST data. For each cell type, we selected ten well‐established marker genes and computed the correlation coefficient between their spatial expression and the corresponding cell type proportion estimated by each method. SPADE had the overall highest rank of correlation coefficient among all methods, supporting the biological interpretability of SPADE's cell type mapping (Figure S23).

The ability of SPADE to enhance gene expression values in spatial locations is especially valuable for high‐resolution ST datasets with low sequencing depth. In the mouse cerebellum dataset, SPADE successfully recovered the expression of key cell‐type‐specific genes, as validated by the co‐localization of cell types and their reconstructed expression (Figure 6d). For example, *Slc1a3*, a marker gene of Bergmann glia cells, was predominantly expressed in the Bergmann glia region [52]. Similarly, *Car8* was specifically expressed in the Purkinje region [53].

The utility of enhancing expression data was further demonstrated by the detection of SVGs that had very low expression measurements in the original ST data (Figure S24). One example is *Cbln2*, which is known to be expressed primarily in choroid interneurons and plays a role in synapse formation and modulation [54]. Another is *Tnc*, which is highly expressed in Bergmann glial cells in the mouse cerebellum, contributing to cell migration, synaptic modulation, and extracellular matrix organization during the developmental stage [55]. These results demonstrate SPADE's ability to recover functionally relevant spatial gene patterns.

To assess the accuracy of gene expression enhancement, we performed an in silico gene imputation study (Figure 6e). We randomly masked 20 highly expressed genes in the original ST data and applied SPADE and other methods currently available for reconstructing gene expression profiles to predict the expression of these masked genes. SPADE, Tangram, and novoSpaRC had similar RMSEs, all of which were lower than Seurat and SpaOTsc, demonstrating their favorable performance in gene expression imputation (Figure 6f). Unlike SPADE, Tangram and novoSpaRC failed to accurately annotate spot types, suggesting potential overfitting to gene expression similarity in these algorithms.

## 3 Discussion

Here, we introduce SPADE, a computational framework for spatial architecture reconstruction and quantitative characterization of CCC by estimating cell type composition and enhancing gene expression patterns using a reference scRNA‐seq dataset and integrating biological knowledge about CCC patterns. SPADE first performs a low‐dimensional embedding to extract gene combinations informative of cell‐type identity. It then scores the cell–cell communication by quantifying the expression of ligand and receptor genes. Finally, SPADE aligns single cells from the scRNA‐seq dataset to spatial locations by jointly minimizing the distance between low‐dimensional embeddings of the original and reconstructed gene expression profiles, while maximizing the concordance between CCC scores and physical proximity. SPADE achieves accurate cell‐type mapping and reconstructs ST profiles with higher gene count and transcript depth, which reliably captures spatial architecture in both well‐structured and spatially heterogeneous tissues. Within the complex tumor microenvironment, SPADE simultaneously resolves intratumoral heterogeneity and delineates distinct malignant domains, while precisely charting the distribution of immune and stromal cell subsets, which improves the understanding of tumor evolution and mechanisms of therapeutic resistance. Besides, through quantification of CCC intensity across spots, SPADE identifies region‐specific CCC events correlated with clinical outcomes in a heterogeneous tumor environment.

SPADE offers several key advantages over existing deconvolution methods. First, the deep‐learning module for dimension reduction in SPADE offers a refined approach to feature selection and noise cancellation. Gene expression profiles in ST datasets are often noisy and sparse, especially in high‐resolution datasets. Some algorithms, such as Tangram, require users to specify marker gene lists for downstream mapping, and thereafter discard all other genes. Although this strategy improves computational efficiency, it may fail to capture the full spectrum of cell type‐specific features. At the same time, calculating expression similarity using the full gene expression spectrum can introduce noisy signals. SPADE addresses this challenge by incorporating an embedding module that combines an autoencoder and a discriminator to identify gene combinations that optimally balance cell type‐discriminative information with noise suppression. This latent‐space embedding not only improves feature representation but also reduces computational demands, since computational complexity scales with the size of the gene expression matrix. Second, some existing methods delete some cell types from assignment consideration based on the reference scRNA‐seq data. For example, RCTD filters out cell types with limited cell numbers in the scRNA‐seq dataset, and Seurat excludes all cell populations that are not designated as “anchors” during integration. However, accumulating evidence indicates that rare cell types can play essential biological roles in tissue organization and disease progression [56]. SPADE retains the features of all cell types to enable a more comprehensive characterization of cellular distributions in ST data. Third, SPADE effectively addresses the limited gene coverage of ST data by mapping the high‐depth gene expression from single cells to spatial locations. The probabilistic mapping mitigates dropout effects by calculating the weighted sum of the expression among all relevant single cells, thereby significantly reducing the probability of zero expression.

A distinctive strength of SPADE is its innovative incorporation of explicit cell–cell communication quantification between single cells and spatial spots to guide the alignment process, based on the assumption that most CCC events occur between spatially proximate cells. We demonstrated that CCCs were intrinsic features of tissue organization (Figure 2b), and that integrating CCC‐based distance constraints improved alignment accuracy. Existing studies measuring single‐cell CCCs often simply multiply the expression of ligand and receptor genes and sum the products across all ligand–receptor pairs to obtain the CCC score between two cells [57, 58]. This strategy often produces inaccurate representations, as only a small subset of ligand–receptor pairs mediates actual intercellular interactions. To solve this problem, SPADE introduces a specificity weight and a regulatory weight to describe the likelihood that a given ligand–receptor pair participates in CCC events, thereby improving the biological relevance of CCC scores. SPADE also offers a unique framework for quantifying CCC at the single‐spot level, allowing users to flexibly define regions of interest and identify their specific ligand–receptor pairs. In the CRCLM dataset, we observed significant differences in CCC events between two tumor subregions, which revealed intratumoral heterogeneity and offered critical insights for targeted clinical treatments. This finding highlights the power of SPADE to overcome the limitations of initial cell‐type annotations from reference scRNA‐seq datasets, enabling a more refined differentiation of cellular subpopulations and their spatial distributions. Moreover, this result may represent another biological principle uncovered by incorporation of CCC constraints into the alignment process—different cellular subpopulations may engage in distinct CCCs, which could in turn influence their spatial localization.

While SPADE is a powerful tool for integrating ST and scRNA‐seq datasets, there is room for improvement. First, the CCC scoring strategy may be further refined. Most available CCC scoring methods are designed at the level of single cells for cell‐type analysis, making them too computationally intensive in ST data optimization. Developing a more streamlined and effective CCC scoring strategy could further enhance the performance of SPADE. Second, our method does not currently account for the potential role of long‐range CCC events, for example, those mediated by some secreted chemokines to recruit remote immune cells. Incorporating these interactions into SPADE could provide a more comprehensive view of intercellular communication and further improve its biological relevance and accuracy in spatial mapping. Third, the mapping performance of SPADE is heavily constrained by the quality of the reference scRNA‐seq dataset, as SPADE still relies on features extracted from the annotated cell types that are derived from the scRNA‐seq reference. Although we have demonstrated that SPADE can mitigate some issues arising from missing or ambiguously annotated cell types, low‐quality or poorly annotated references can still degrade mapping accuracy. This highlights the need for users to carefully select well‐matched, high‐quality references prior to analysis. Additionally, the optimization module of SPADE may require substantial computational resources for very large datasets. To address this, we plan to develop a simplified model that restricts CCC scoring to neighboring spots within a defined threshold, thereby reducing computational demand without compromising performance.

Besides, we note that benchmarking on real datasets is inherently limited by the absence of ground‐truth cell mapping. In these analyses, concordance with RCTD should be interpreted as agreement with an established reference method, rather than as a direct measure of mapping accuracy. RCTD itself is subject to its own modeling assumptions and potential biases. In the last application, we compared the result from each method with the majority consensus. While this strategy provides a practical consensus‐based comparison, it may introduce bias toward methods that exhibit higher correlations with others, potentially disadvantaging less correlated algorithms. Evaluation on simulated datasets, where ground‐truth assignments are known by construction, provides a more rigorous assessment of absolute accuracy. Comparisons on real datasets serve as a complementary perspective, demonstrating consistency with existing methods under realistic conditions.

A major goal of spatial transcriptomics is to characterize the cellular context and tissue architecture. SPADE estimates cell‐type composition and enhances the gene expression data, enabling more accurate and detailed spatial characterization. By incorporating cell–cell communication events into the spatial alignment process, SPADE improves the biological relevance of the inferred spatial maps and provides a more faithful representation of the local tissue microenvironment, informing potential therapeutic interventions. As ST technologies continue to advance and more datasets become available, we expect that SPADE will be used broadly to characterize tissue and cellular organization.

## 4 Methods

### 4.1 SPADE Workflow

#### 4.1.1 Data Preparation

The SPADE framework requires two inputs: a spatial transcriptomics (ST) gene expression matrix *Y* with spot coordinates and a matched single‐cell gene expression matrix *X*. Cell annotations $A=(a_{1},\;a_{2},\text{…},\;a_{n})$ of scRNA‐seq data are preferable for enabling the full SPADE model. Users can also provide a custom L–R pair database in place of the default CellChatDB database. To avoid unbalanced cell type proportions in the single‐cell dataset, SPADE performs down‐sampling for cell types whose proportion exceeds three times the average proportion across all cell types, reducing it to exactly threefold the average.

#### 4.1.2 Marker Gene Identification

For single‐cell datasets having cell annotations, SPADE first identifies the marker genes for each cell type. Marker genes should satisfy three criteria: (1) the mean expression in the given cell type should be at least twice than that in other cell types, that is, fold‐change > 2; (2) at least half of the cells of the given cell type have positive expression; (3) the adjusted *p*‐value should be smaller than 0.05. We select the top 150 genes ordered by fold‐change for each cell type. Differential expression (DE) analysis is performed by the FindAllMarkers() function from Seurat. For datasets without cell annotations, top 3000 Highly Variable Genes (HVGs) are extracted as marker genes. Only marker genes are considered as cell features in the following.

#### 4.1.3 Single‐Cell Latent Space Embedding

The goals of the dimension‐reduction model are: (1) removing technical and biological noise while retaining essential biological signals; (2) identifying key gene modules or principal components that capture most of the variance in the data; (3) maximizing separability of different cell types while minimizhin‐class variances.

First, the single‐cell gene expression matrix is normalized to have identical column sums to remove the influence of library size of different cells. For convenience, we denote the normalized full expression matrix as *X*_full_ and the normalized marker gene‐by‐cell matrix as *X*.

The emdedding model comprises an autoencoder module and a discriminator module. The encoder consists of two fully connected layers: the first fully connected layer employs 512 neurons, and the second fully connected layer reduces the number of neurons to 128, each followed by a ReLU activation function, creating a bottleneck layer of 128 dimensions. The decoder module employs the two fully connected layers with 128 and 512 neurons, respectively. The bottleneck layer $Z\;\in \;R^{64*n_{\mathrm{cell}}}$ is connected to the discrimination module, and we denote *Z*  =  *f*(*X*), where *f* represents the encoder function. To describe the performance of the autoencoder, we use MSELoss as the reconstruction error, denoted as *L*_re_.

The discrimination module serves as a supervised learning program for cell features, which maximize the distance between different cell types while minimizing the within‐class variations. The total within‐class variation is calculated as:

$$S_{w}=\sum_{c}\sum_{i:a_{i}=\;c}\frac{1}{n_{c}}∥Z_{i}-\mu_{c}{∥}^{2}\;,$$

where *n_c_* stands for the number of cells belonging to cell type *c*, and μ_*c*_ stands for the mean embedding vector of all cells belonging to type *c*. The distance between cell types is calculated as:

$$S_{d}=\sum_{c_{1}}\sum_{c_{2}\neq c_{1}}∥\mu_{c_{1}}-\mu_{c_{2}}{∥}^{2}\;,$$

where *c*_1_ and *c*_2_ are two different cell types. We define the discrimination loss as the ratio of within‐class variations to between‐class distance:

$$L_{d}\;\left(\theta \right)=\frac{S_{w}}{S_{d}}.$$

The total loss function, which is proposed to reduce the reconstruction error in the autoencoder module while increasing the discriminability of cell types, is described as follows:

$$L\;\left(\theta \right)=L_{\mathrm{re}}\;\left(\theta \right)+\lambda L_{d}\left(\theta \right),$$

where the first term of this equation controls the feature extraction accuracy, whereas the second term controls the feature specificity. θ represents the model parameter, and λ is a hyperparameter to adjust the weights of the two loss terms.

For a single‐cell dataset without cell annotations, *L*_re_(θ) is used as the total loss function. Model training is performed via the Adam optimizer using the PyTorch library.

#### 4.1.4 Cell–Cell Communication Scoring for Single‐Cell Datasets

We develope a computational model to describe the probability of cell–cell communication (CCC) events between any two single cells. More than 2000 ligand–receptor (L–R) gene pairs are collected in CellChatDB. Not all L–R pairs contribute equally to the CCC events, so we designe a weighting algorithm to describe the contribution of L–R pairs for CCC events. L–R pairs that are informative for spatial mapping should reflect cell‐specific or cell‐type‐specific communication rather than ubiquitous signaling. L–R pairs with cell‐specific expression define unique communication relationships between particular cell populations, providing stronger constraints on spatial co‐localization. Since we require both the ligand and the receptor to be cell‐specific simultaneously, we define the weight *w* measuring the specificity of the L–R pairs as described below. The genes exhibiting significantly higher expression levels within a restricted subset of cells have higher specificity weight. The specificity is quantified through the calculation of entropy for each ligand and receptor gene, defined as:

$$H\left(g\right)=-\sum_{j=1}^{n}\frac{x_{g,\;j}}{X_{g}}\log\frac{x_{g,\;j}}{X_{g}},$$

where *n* is the number of cells in this dataset, *x*_*g*, *j*_ is the expression of gene *g* in cell *j*, and *X_g_* is the sum of *x*_*g*, *j*_ for all cells. The weight *w* of a given L–R pair is then given by:

$$w\;\left(L-R\right)=\frac{1}{1+H\left(L\right)+H\left(R\right)}.$$

Next, we define the CCC score between two cells. For each L–R pair, we quantify the geometric mean of the ligand and receptor gene expression values and then normalize across all cell pairs:

$$\;{\hat{s}}_{ij}=\frac{\left(s_{ij}-\bar{s}\right)}{\sigma },$$

where *s_ij_* is the geometric mean of ligand and receptor expression of cell *i* and *j*, $\bar{s}$ and σ is the average and standard deviation of *s_ij_* among all cell pairs, respectively. We drop the negative values after normalization, and cap the ${\hat{s}}_{ij}$ at a threshold (5 by default) to mitigate the influence of exceptionally high‐expressing genes.

SPADE then measures the probability that CCC events occur through the given L–R pairs. The simultaneous high expression of the ligand and receptor genes does not necessarily indicate CCC events. This is because the ligand and receptor genes may be simultaneously overexpressed by chance. We hypothesize that the activation of CCC through an L–R pair is modulated by the activity of the corresponding transcription factors (TFs). To quantify this, we calculate the correlation coefficients between the expression of the gene and those of its regulatory TFs across all cells:

$$\;r_{g}=\frac{1}{l}\;\sum_{h}\left|\mathrm{cor}\left(x_{h},\;x_{g}\right)\right|,$$

where *h* represents the TFs of the given gene *g* in the database, *l* is the number of TFs of the given gene *g*, and *x_g_* is the gene expression vector across all cells.

Collectively, the CCC score between cell *i* and cell *j* is defined as:

$$\;I_{ij}=\sum_{L-R}w\left(L-R\right)*{\hat{s}}_{ij}*r_{L}*r_{R}.$$

#### 4.1.5 The Mapping Algorithm

We use the index *i* for single cells, *k* for embedding features, and *j* for spatial spots. The mapping algorithm is to learn an alignment to spatial locations from the single cells, namely a mapping matrix $W\in R^{n_{\mathrm{cell}}*n_{\mathrm{spot}}}$, where *n*_cell_ is the number of single cells and *n*_spot_ is the number of spatial spots. SPADE aims to learn *W* such that $W_{ij}\in \left[0,\;1\right]$ is the probability of single cell *i* mapping to spatial location *j*. SPADE requires the constraint $\sum_{i}W_{ij}=1$ to ensure all spots have the same gene count.

Goals of mapping include: (1) the predicted gene expressions should be similar with the original gene expressions, and (2) single cells connected by CCC events should be in close proximity to each other. We denote $\hat{Z}=ZW$ as the predicted embeddings from the mapping matrix. SPADE calculates the similarity between the predicted embeddings $\hat{Z}$ and the original expression embedding *f*(*Y*) using cosine similarity. To harmonize the CCC scores and the distance between two cells, SPADE performes the transformation of CCC scores and the distance. We define the transformation function as following:

$$h\;\left(X\right)=\mathrm{sigmoid}\left(\frac{X-\mathrm{mean}\left(X\right)}{2\mathrm{std}\left(X\right)}-1\right),$$

where mean() and std() functions calculate the mean and standard deviation of the elements in the matrix, and the sigmoid() function is defined as:

$$\mathrm{sigmoid}\;\left(x\right)=\frac{1}{1+e^{-x}}.$$

To learn the mapping matrix, SPADE minimizes the following loss function with respect to *W*:

$$\begin{array}{ccc} L\;\left(W\right) & = & -\sum_{k=1}^{n_{\mathrm{embed}}}\cos\left(f{\left(Y\right)}_{k,*},{\hat{Z}}_{k,*}\right)-\sum_{j=1}^{n_{\mathrm{spot}}}\cos\left(f{\left(Y\right)}_{*,j},{\hat{Z}}_{*,j}\right)\\ & & -\lambda_{1}\sum_{j=1}^{n_{\mathrm{spot}}}\cos\left(h{\left(-D\right)}_{*,j},\;h{\left(W^{T}IW\right)}_{*,j}\right)-\lambda_{2}\mathrm{tr}\left(W^{T}\log W\right), \end{array}$$

where *D* represents the distance matrix between spots, Cos() is the cosine similarity function, * indicates matrix slicing, tr() is the trace of matrix, λ_1_ and λ_2_ are hyperparameters.

Minimization is obtained via gradient‐based optimization using the PyTorch library. The non‐negative constraint of mapping matrix *W* is achieved by the softmax transformation:

$$W_{ij}=\mathrm{softmax}\;{\left(\tilde{W}\right)}_{ij}=\frac{e^{{\tilde{W}}_{ij}}}{\sum_{l}e^{{\tilde{W}}_{lj}}},$$

where $\tilde{W}$ is the training variable in the optimization.

#### 4.1.6 Cell Type Composition Estimation

For single‐cell datasets with cell annotations, SPADE estimates cell type composition for each spatial spot by the mapping matrix *W* and cell type indicator matrix *T*. Here, *T* is a zero‐one cell type‐by‐cell matrix, where *T_ij_*  =  1 indicates cell *j* is annotated to cell type *i*. SPADE estimates the cell type abundance by *C*  =  *TW*. Note that since the column sums of *T* and *W* equal to 1, the column sums of *C* also equal to 1. SPADE discards the tiny cell types whose abundance is less than 0.0001, as they appear to be the mapping noises.

#### 4.1.7 Spatial Gene Expression Enhancement

In the ST data, the total gene count of each spot can be varied significantly due to the different number of cells in each spot or the difference in RNA capture efficiency. SPADE generates an enhanced gene count matrix with an identical sum of gene counts in each spot, and the expressions are proportional to the weighted sum of the expressions of single cells. The enhanced gene expression of each spot is calculated as:

$$\;\hat{Y}=\mathrm{round}\left(\gamma X_{\mathrm{full}}W\right),$$

where γ represents the mean gene counts among the spots and the round() function rounds all numbers to integers. Note that since the column sums of *X*_full_ and *W* equal to 1, the matrix $\hat{Y}$ has identical column sums of γ.

#### 4.1.8 CCC Activity Estimation for Spots

SPADE calculates the CCC score *I_ij_* between spots *i* and *j* by treating spots as single cells and applying the same method in the “cell–cell communication scoring for single‐cell datasets” section. To reveal and visualize the CCC activity for each spot, SPADE calculates the Gaussian kernel‐weighted sum of CCC scores between the target spot *i* and all other spots:

$$\;A_{i}=\sum_{j}I_{ij}e^{-\frac{d_{ij}^{2}}{\sigma^{2}}},$$

where *d_ij_* represents the distance between spot *i* and *j*, and σ indicates the bandwidth of the Gaussian kernel (equal to the distance between neighboring spots by default).

#### 4.1.9 Detection of Active L–R Pairs in a Given Region

SPADE detects region‐specific L–R pairs by calculating the CCC score of the given L–R pair between spots in the given region and out of the given region. *t*‐test is performed to report the L–R pairs having a significant difference between in and out of the region.

### 4.2 Simulation Study

#### 4.2.1 Generation of Simulated Data

We selected a real intestinal ST dataset to be the template of our simulated datasets. We also downloaded a single‐cell dataset sequenced from an intestinal tissue as a matched reference dataset. The main steps of the generation of simulated datasets include: (1) determine the cell type proportion of each spot in the ST dataset; (2) assign single cells to the spatial spots and generate the gold standard of cell type abundance; (3) calculate the gene expression of each spot in the simulated datasets according to the assignment.

We first estimated the cell type proportion of each spot using RCTD with its default parameters. We set the number of single cells per spot to 10. Next, to assign a specific number of cells for each cell type at a given spot, we sampled from a multinomial distribution with the probability vector equal to the cell type proportion from RCTD:

$$c_{j}∼\mathrm{Multinomial}\left(10,p_{j}\right),$$

and where *c_j_* was the number of cells for each cell type assigned to the spot *j*, and *p_j_* was the cell type proportion estimated by RCTD at the spot *j*. $p_{j}^{\mathrm{true}}≔\frac{c_{j}}{10}$ was the true cell type proportion in each spot. We randomly selected the corresponding number of single cells in each cell type and calculated the sum of their gene expression defined as μ_*j*_. Similar to the model design, the single‐cell gene expression matrix was pre‐processed to be normalized to identical column sums.

We then generated gene expressions for each spot. To mimic the distribution of gene counts in the real dataset, the sum of gene expressions in each spot was set to be approximately the same as its original total gene count. Specifically, we scaled the gene expression vector μ_*j*_ to have the same sum of its original total gene count, and sampled the gene expression *e_kj_* from a zero‐inflated negative binomial (ZINB) distribution:

$$e_{kj}∼\mathrm{ZINB}\left(\frac{\mu_{kj}}{1-\pi_{k}},\phi ,\;\pi_{k}\right),$$

where π_*k*_ was the probability of zero expression of gene *k* and ϕ was the over‐dispersion parameter set to 0.2.

Finally, we added dropout events and spot swap events as sequencing noises to the gene expression matrix. The expression was set to zero if a dropout event occurred, where the dropout rate was set to $0.01e^{-\frac{\mu_{j}^{2}}{4}}$. In spatial transcriptomics technologies such as 10× Visium, RNA molecules can diffuse across adjacent capture spots during tissue permeabilization and library preparation, resulting in a fraction of transcripts being captured at a neighboring spot rather than their true location of origin. This spatial cross‐contamination is more likely to occur between spots that were physically closer to each other. To simulate this technical noise, we introduced spot swap events, where the expression of a gene at spot 1 was replaced by the expression from a neighboring spot 2 with a probability proportional to their physical proximity. The spot swap probability was set to $0.001e^{-\frac{d_{j_{1}j_{2}}^{2}}{d_{0}^{2}}}$, where $d_{j_{1}j_{2}}$ was the distance between spot *j*_1_ and *j*_2_, and *d*_0_ was the minimum distance between any two spots.

In addition, to evaluate the contribution of cell–cell interaction in the mapping, we designed another simulated dataset. We randomly selected three cell types and split the ST slice into two parts, where cell types A and B were distributed only in the upper and lower parts, respectively. Cells in cell type C were randomly divided into two subgroups, namely cell type C1 and C2, which were also distributed only in the upper and lower parts of the slice, respectively. We randomly selected 20 ligand–receptor gene pairs from CellChatDB. We then elevated the expression of the first ten ligand and receptor genes in cell type A and C1, respectively, and elevated the expression of the other ten ligand and receptor genes in cell type B and C2, respectively. The expression elevation was performed by sampling from a negative binomial (NB) distribution:

$$e_{ki}∼\mathrm{NB}\left(3\mu_{k},\phi \right),$$

where μ_*k*_ was the mean expression of gene *k* and ϕ s the over‐dispersion parameter set to 0.2.

#### 4.2.2 Generation of Simulated Data Using scMultiSim

We used scMultiSim, an R package designed to simulate single‐cell multi‐omics and spatial transcriptomics data with user‐specified CCC patterns. For each simulated dataset, we randomly selected 50 active L–R pairs and assigned the CCC effect scores as exp (*U*), where *U* followed a uniform distribution ranging between 0 and 2. We generated 3600 single cells arranged on a 60 × 60 square grid with 10 cell types. To mimic spatial transcriptomics resolution, we aggregated cells within non‐overlapping 3 × 3 bins into individual spots, resulting in a spatial transcriptomic dataset containing 20 × 20 spots. We also added some sequencing noise using the add_expr_noise() function to mimic the real data distribution.

#### 4.2.3 Benchmark Settings

When SPADE was applied to real‐world datasets, users might not provide perfectly matched single‐cell reference datasets. Therefore, evaluated the performance of SPADE under unsatisfactory conditions. We designed three scenarios to evaluate the performance of SPADE.

Scenario 1: the cell types in the single‐cell reference dataset perfectly matched those in the ST dataset. This was an ideal situation under which the algorithms should have the best performance.

Scenario 2: one cell type in the ST dataset was missed in the single‐cell reference dataset. We removed all the cells of a randomly selected cell type from the single‐cell dataset to mimic the situation of cell type mismatch.

Scenario 3: two cell types were mixed in the single‐cell reference dataset. We randomly selected two cell types and shuffled the labels of cells of these two cell types to mimic the situation that cell subtypes were mis‐assigned.

### 4.3 Benchmark Metrics

To evaluate the performance of SPADE and other algorithms, we used five metrics: root mean square error (RMSE), major type accuracy, major 2 type accuracy, expression correlation, and subtype error proportion.

1. RMSE: A metric that is proportional to the Euclidean distance of the true cell type proportion vector and the predicted cell type proportion vector:
$$\mathrm{RMSE}=\sqrt{\frac{1}{n_{\mathrm{type}}}{\left(p_{j}^{\mathrm{true}}-p_{j}^{\mathrm{pre}}\right)}^{2}},$$

1. Major type accuracy: the proportion of spots that the predicted major type matches the true major type. Here, major type represents the most abundant cell type in one spot.
2. Major 2 type accuracy: the proportion of spots that the predicted two major types match the true two major types. Here, two major types represent the top 2 abundant cell types in one spot, disregarding the order.
3. Expression correlation: the mean of Pearson's correlation between the enhanced expression and original expression.
4. Subtype error proportion: the proportion of cell type C1 and C2 mapped to the wrong part of the slice.

where $p_{j}^{\mathrm{pre}}$ is the predicted cell type proportion at spot *j* and *n*_type_ is the number of cell types in the reference dataset.

### 4.4 Method Comparisons

We compared SPADE with nine other algorithms, including RCTD, Tangram, CytoSPACE, Seurat, SpaOTsc, Cell2location, novoSpaRc, SpatialDWLS, and SPOTlight. For RCTD, we used the “full” mode for all datasets. For Tangram, we set the parameters as modes = ‘clusters’, density = ‘rna_count_based’. For Seurat, we followed the instructions on the Seurat website (https://satijalab.org/seurat/). For other algorithms, we followed the corresponding tutorials and used the recommended default parameter settings.

### 4.5 Mouse and Human Data Analysis

For the mouse brain dataset, we used the deconvolution result of RCTD as the reference standard of cell type distribution, and RNA in situ hybridization (ISH) data from the Allen Mouse Brain Atlas (https://mouse.brain‐map.org) as the ground truth of gene expression in space. After deconvolution of different methods, we computed the K–L divergence between each deconvolution result and the RCTD reference using the KLD() function from the LaplacesDemon R package (v 16.1.6). For the original and SPADE‐reconstructed spatial transcriptomics data, we applied standard SCTransform() normalization and identified SVGs along with their Moran's I values using the RunMoransI() function in the Seurat R package (v 5.0.1). For the DLPFC dataset with annotations of cortical layers, to evaluate the deconvolution precision of each method, we calculated the proportion of each cell type's predicted spatial distribution that was correctly mapped to its corresponding cortical layer. A higher precision score indicates more accurate spatial mapping. The SVG identification procedure followed the same pipeline of the mouse brain dataset.

In the TNBC dataset analysis, mapping accuracy was evaluated by Pearson's and Spearman's correlation between inferred cell‐type proportions and expression of corresponding cell‐type‐specific marker genes across spatial locations for each algorithm. The cell‐type signature list was from the original article. SVGs were identified by Hotspot (v 1.1.1) with default parameters, applying selection criteria of FDR < 0.001 and *p*‐value < 0.01. We performed GO pathway enrichment analysis through the enrichGO() function in the clusterProfiler R package (v 4.6.2), and declared pathways to be significantly enriched on the basis of a BH‐adjusted *p*‐value threshold of 0.05. The pathway and TLS scores were calculated by the AddModuleScore() function in the Seurat R package.

For the CRCLM dataset, cell‐type colocalization was evaluated by Pearson's correlation among cell type proportions across the slide. We performed unsupervised clustering of SPADE‐reconstructed spatial transcriptomic profile following the standard Seurat pipeline. We annotated the clusters based on the histological annotations described in the original study. Specifically, the spatial architecture was characterized by normal liver parenchyma on the left and scattered colorectal cancer foci embedded within the stroma on the right. The cluster located at the interface between these two domains was marked as the Border region. We then validated the annotations according to the spatial expression pattern of three canonical marker genes from the original study, as the Normal liver region was characterized by robust *ALB* expression, the Stroma region was marked by *COL1A1*, and the Tumor regions were defined by high *EPCAM* expression. The HVGs of each region were identified through the FindMarkers() function in the Seurat package. For the tumor region, five subregions were designated as ident.1, and all other regions were used as ident.2. GO and KEGG pathway analysis were performed through enrichGO() and enrichKEGG() functions in the clusterProfiler R package with pvalueCutoff = 0.05 and qvalueCutoff = 0.05. SMCC subtype scores were computed by the AddModuleScore() function of the Seurat R package based on gene signatures reported in the original study. To investigate differential ligand–receptor interactions among tumor cell subtypes in regions T1 and T3, we first used SPADE to identify ligand–receptor pairs significantly enriched in T1 or T3 compared to the background. We then applied the CellChat (v 2.2.0) standard workflow to the reference single‐cell dataset, only retaining significant interactions involving tumor cells as either senders or receivers. The significance threshold of SPADE or CellChat was set at *p*‐value < 0.01 or 0.05, respectively. The final set of ligand–receptor pairs was defined as the overlap of the results from SPADE and CellChat. Chord plots were generated using the chordDiagram() function from the circlize R package (v 0.4.16). Based on histological image features, we manually refined the tumor‐normal boundary. In distance‐based analysis, we calculated the Euclidean distance of each remaining spot to the nearest boundary spot. Spots in the normal region were assigned negative distance values and spots in the tumor side were assigned positive distance values to indicate distance away from the boundary. We declared ligand–receptor pairs to be significantly enriched in the border on the basis of a *p*‐value threshold of 0.05 and a log fold change threshold of 1. GO enrichment analysis of these ligand–receptor pairs was performed as described above.

In the Slide‐seq V2 dataset study, a large number of spots have very low gene counts, which made it hard for SPADE and other methods to estimate the spot type; we marked these spots in grey and ignored them in the subsequent analysis. FindSpatiallyVariableFeatures() from Seurat was used to detect spatial variable genes. The in silico study of gene expression enhancement was performed by first selecting ten genes with total expression count across all spots larger than 3000 and selecting ten genes with total expression count across all spots between 1000 and 3000. We then deleted the selected genes in the ST data and applied methods to obtain the reconstructed gene expression. We compared the difference between original expression and reconstructed expression by calculating the RMSE metric divided by the mean expression of these genes. CytoSPACE encountered an internal error that failed to report the predicted gene expressions.

## Author Contributions

Z.J., N.Z., and X.L. conceived the study. Z.J. and X.L. designed the experiments. Z.J. and X.L. developed the computational method and performed data analysis. Z.J., N.Z., and X.L. wrote the manuscript. Z.J. and N.Z. supervised the work.

## Funding

This work was supported by the National Natural Science Foundation of China (82588201 and 82341005 to N.Z. and 32400510 to Z.J.), the National Science and Technology Major Project of China (2022YFC3400900 to N.Z.), the China Postdoctoral Science Foundation (2022M720308 to Z.J.), and the Beijing Natural Science Foundation (5244030 to Z.J. and QY23058 to X.L.).

## Conflicts of Interest

The authors declare no conflicts of interest.

## Supporting information




**Supporting File 1**: advs76142‐sup‐0001‐SuppMat.pdf.

**Supporting File 2**: advs76142‐sup‐0002‐TableS1‐S8.xlsx.

## Data Availability

The public data used in this work can be accessed through the following links: human intestinal ST (slide A1) and paired scRNA‐seq data for simulation are available at https://simmonslab.shinyapps.io/FetalAtlasDataPortal/; mouse brain ST data by 10X Visium is available at https://support.10xgenomics.com/spatial‐gene‐expression/datasets/1.0.0/V1_Mouse_Brain_Sagittal_Anterior, with reference scRNA‐seq data from the Allen Institute available at https://www.dropbox.com/s/cuowvm4vrf65pvq/allen_cortex.rds?dl=1; human DLPFC ST data (sample 151673) is available at http://research.libd.org/spatialLIBD/, with paired scRNA‐seq from there donors (preprint) available at https://github.com/LieberInstitute/10xPilot_snRNAseq‐human#processed‐data; human TNBC ST data (CID44971) is available at https://zenodo.org/records/4739739#.YY6N_pMzaWC, with paired scRNA‐seq data available at GEO accession GSE176078; human CRCLM ST data (sample P10) is available at GEO accession GSE206552, with reference scRNA‐seq data available at GSE178318; mouse cerebellum ST data by Slide‐seq and reference scRNA‐seq data are available at the Broad Institute Single Cell Portal at https://singlecell.broadinstitute.org/single_cell/study/SCP948. SPADE is available on GitHub (https://github.com/ZijieJin/SPADE).
