# Supplementary material for: SPADE: A Deep Learning Framework for Spatial Mapping and Quantitative Cell–Cell Interaction Inference
Source: Adv Sci (Weinh). 2026 Jun 18:e76142. Online ahead of print. doi: 10.1002/advs.76142 (PMC13336374; doi:10.1002/advs.76142)
Supplement: Supplementary file 1 — Supporting File 1: advs76142‐sup‐0001‐SuppMat.pdf. [file ADVS-9999-e76142-s002.pdf]

SPADE: a deep learning framework for spatial mapping and quantitative cell–cell interaction inference

Xinyi Li[1], Ning Zhang[1,2,3,4,*], Zijie Jin[1,2,5,*]

[1]Department of Immunology, School of Basic Medical Sciences, Health Science Center, Peking University, Beijing, 100191, China

[2]Peking University International Cancer Institute, Health Science Center, Peking University, Beijing, 100191, China

[3]Translational Cancer Research Center, Peking University First Hospital, Beijing 100034, China

[4]Yunnan Baiyao Group Co., Ltd., Kunming, 650500, China

[5]School of Mathematics and Statistics, Beijing Institute of Technology, Beijing, 100088, China

*To whom correspondence should be addressed. Email: zhangning@bjmu.edu.cn and zijiejin@bit.edu.cn

This supplementary file includes:

Supplementary Figure 1 to 24

Supplementary Figures

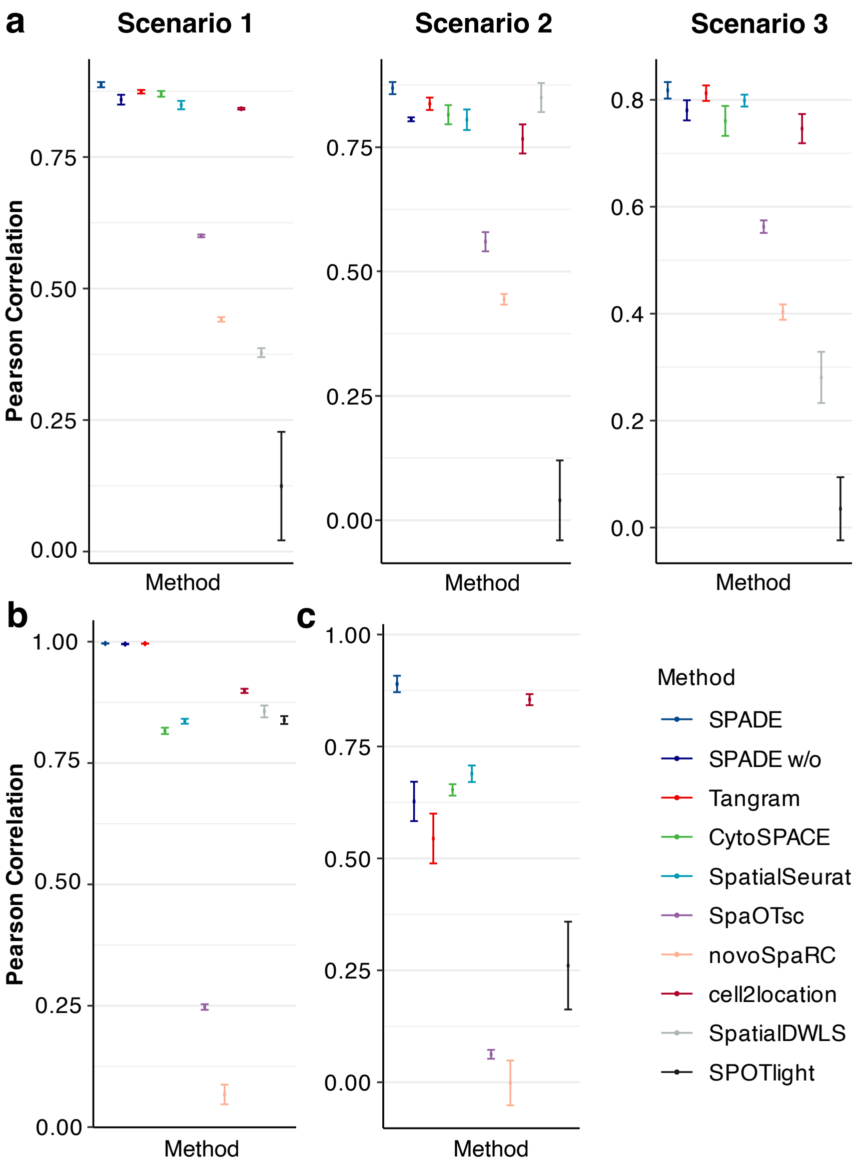

Supplementary Fig. 1. The Pearson's correlation of SPADE and other methods in simulation studies. (**a**) The Pearson's correlation between estimated and true cell type proportion in three scenarios. (**b**) The Pearson's correlation between estimated and true cell type proportion in the simulated dataset generated by scMultiSim. (**c**) The Pearson's correlation between estimated and true cell type proportion in the second additional simulated dataset. The error bar indicates the ± 1 standard deviation of mean accuracy.

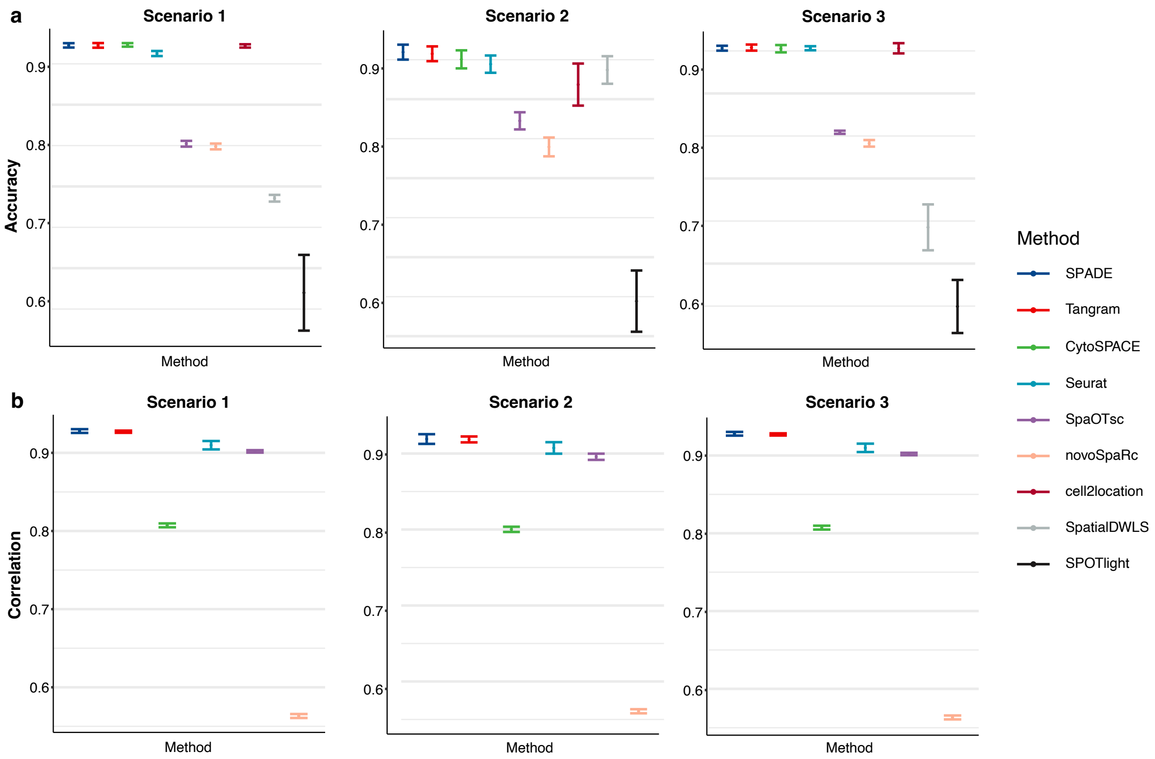

Supplementary Fig. 2. The performance of SPADE and other methods in simulation studies. (**a**) The accuracy of top two dominate cell detection. (**b**) The correlation between original gene expression and reconstructed gene expression across all genes. The error bar indicates the ±1 standard deviation of mean accuracy.

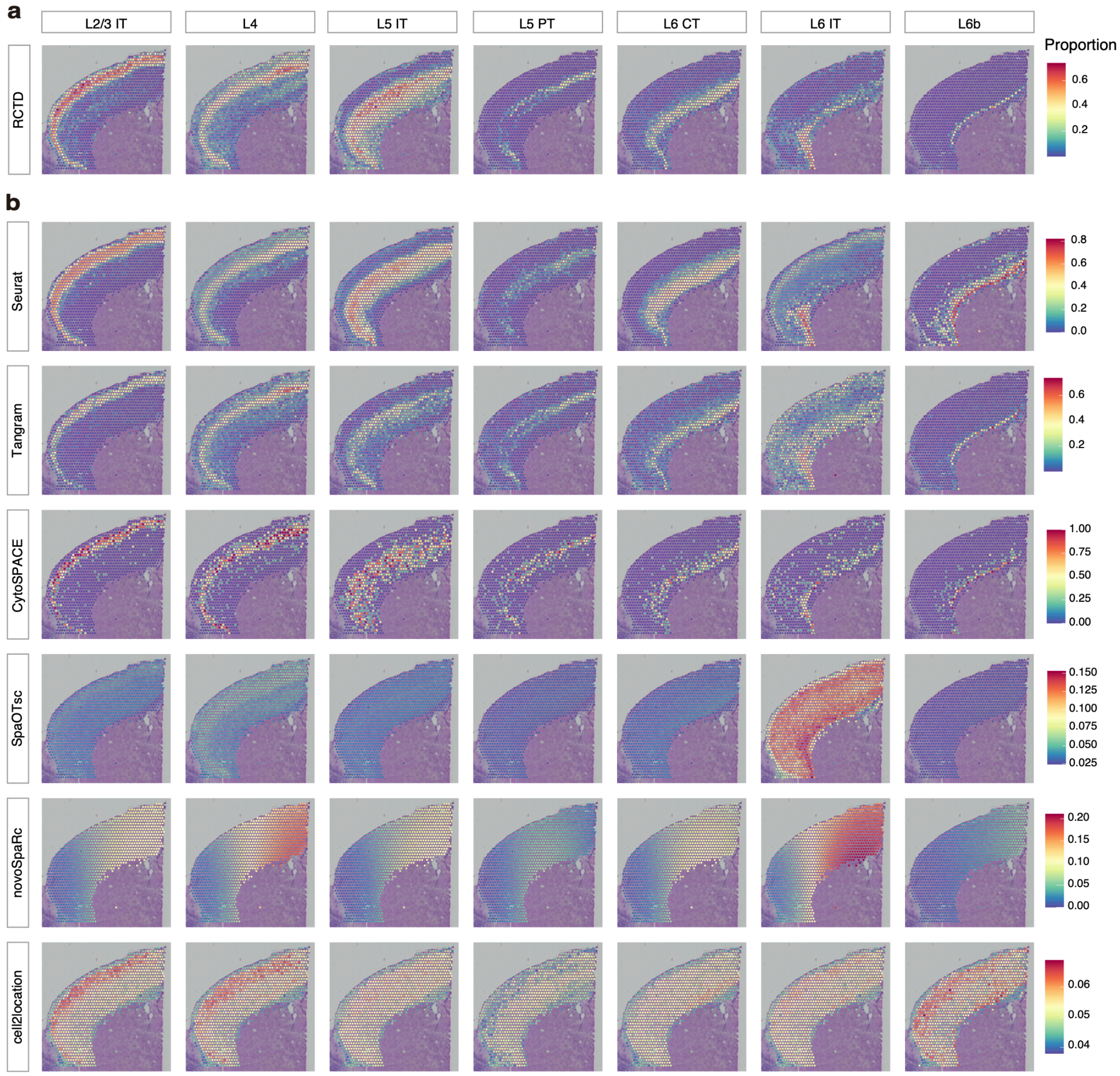

Supplementary Fig. 3. The cell type proportion predicted by deconvolution methods. (**a**) The cell type proportion estimated by RCTD as a reference. (**b**) The cell type proportion estimated by Seurat, Tangram, CytoSPACE, SpaOTsc, novoSpaRc and cell2location.

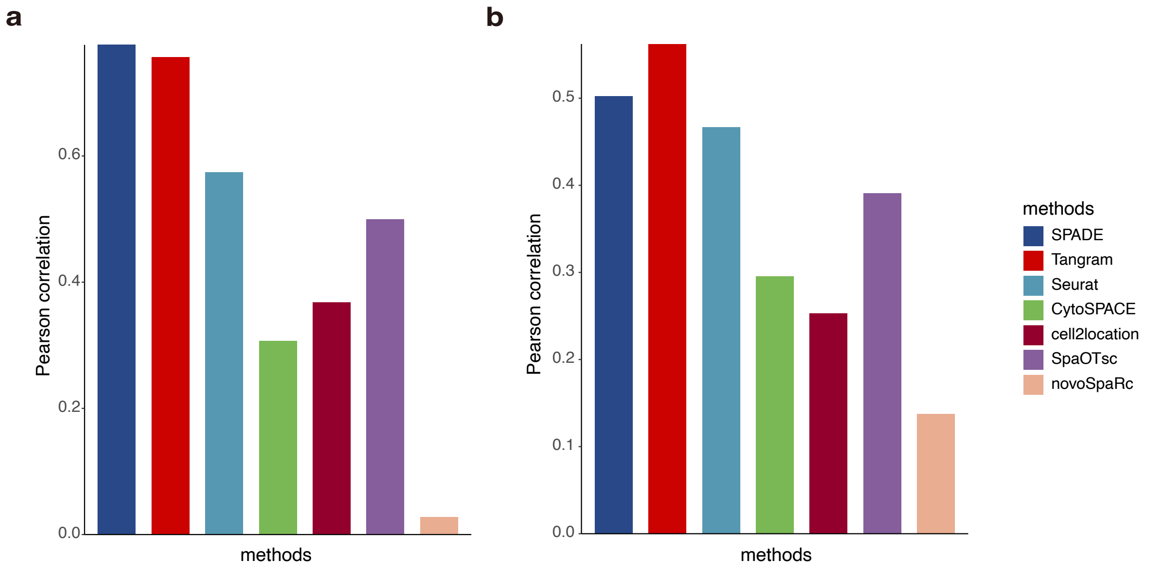

Supplementary Fig. 4. Benchmark using Pearson's correlation on the mouse brain and DLPFC datasets. Mean Pearson's correlation coefficient of cell type alignment by SPADE and other methods on (**a**) mouse brain and (**b**) DLPFC datasets.

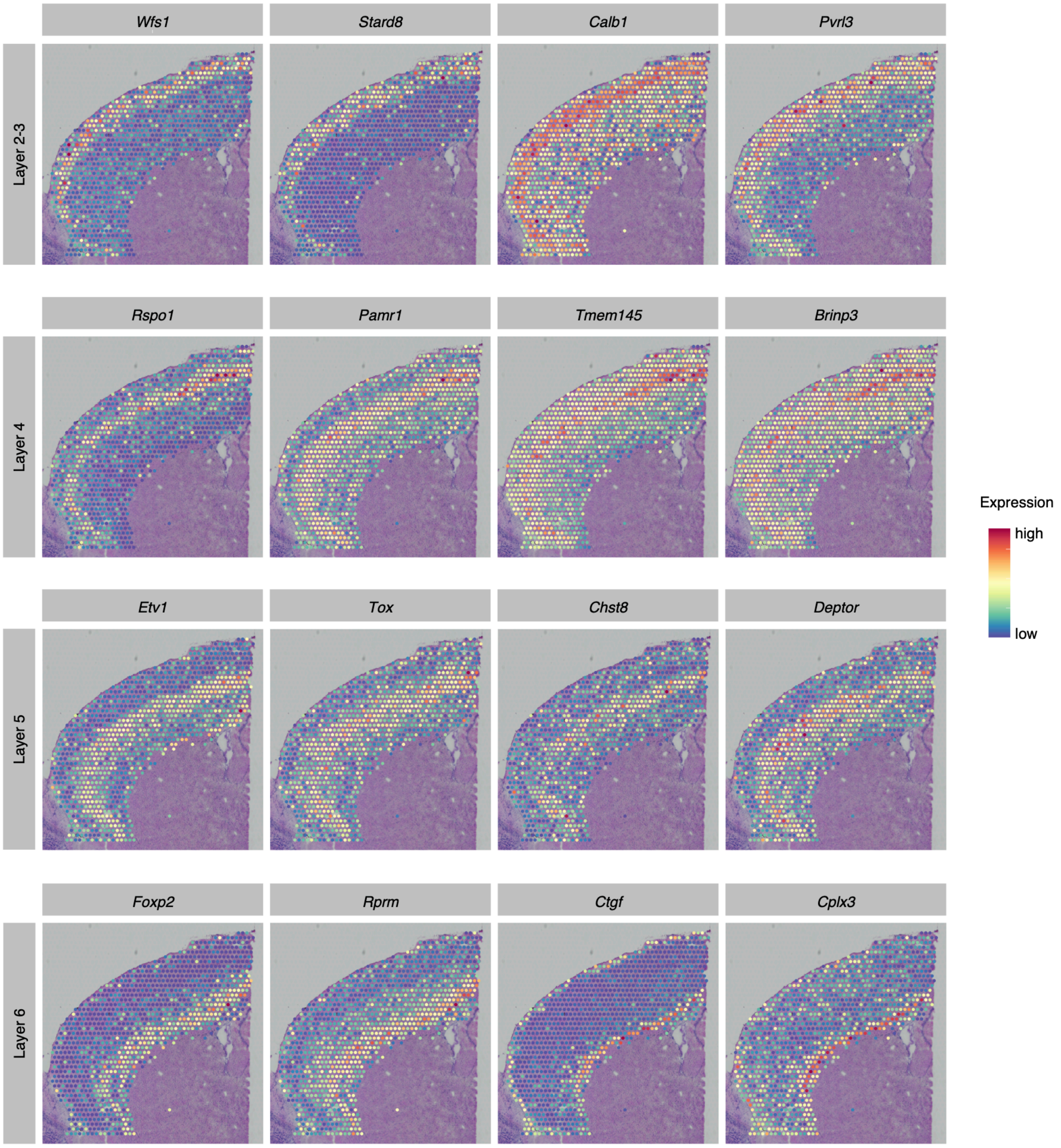

Supplementary Fig. 5. The reconstructed expression of marker genes for each layer by SPADE.

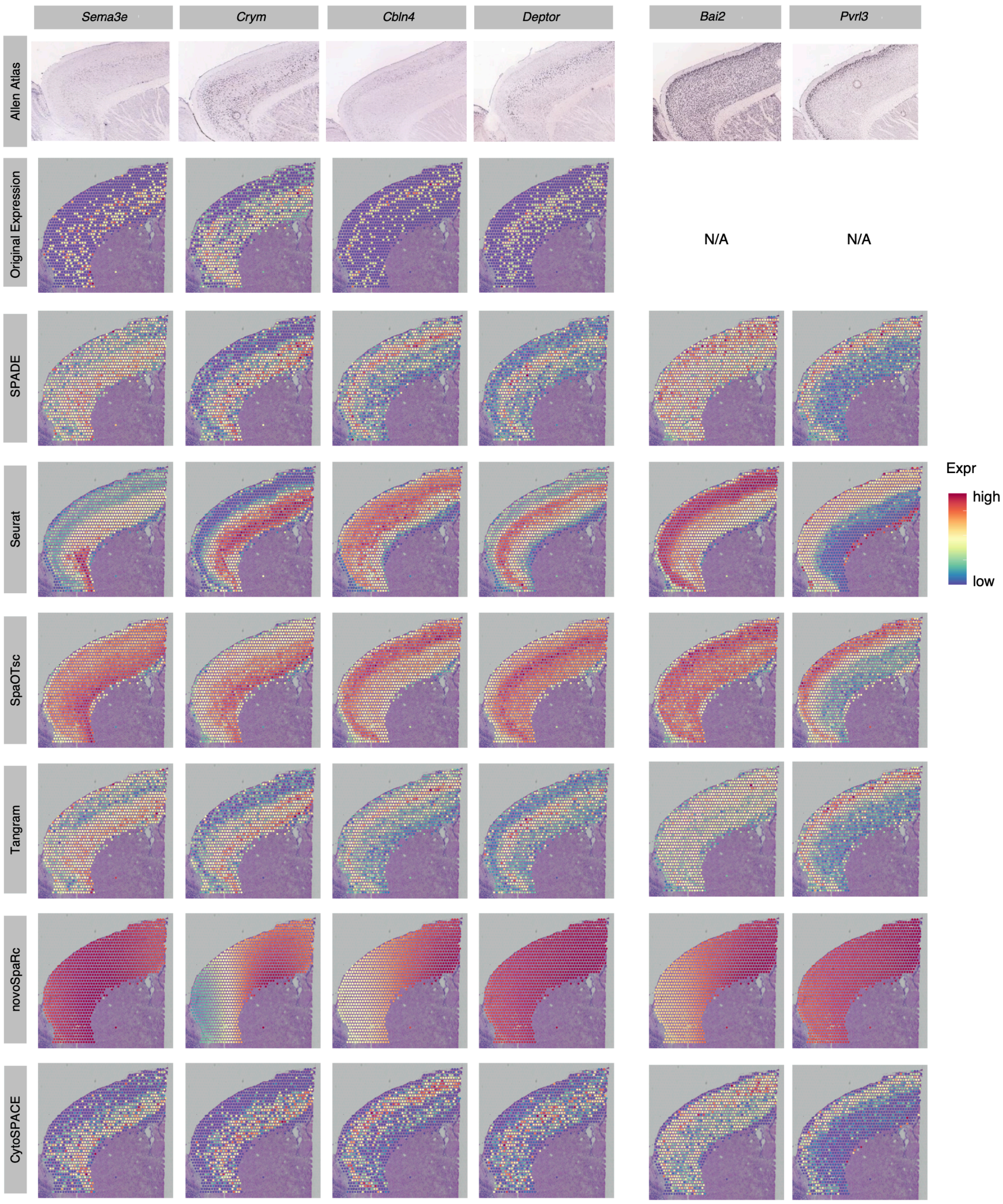

Supplementary Fig. 6. The gene expression enhancement from SPADE and other methods. Top line: the ISH image intensity for the given genes from Allen brain reference atlas. The second line: the original gene expression.

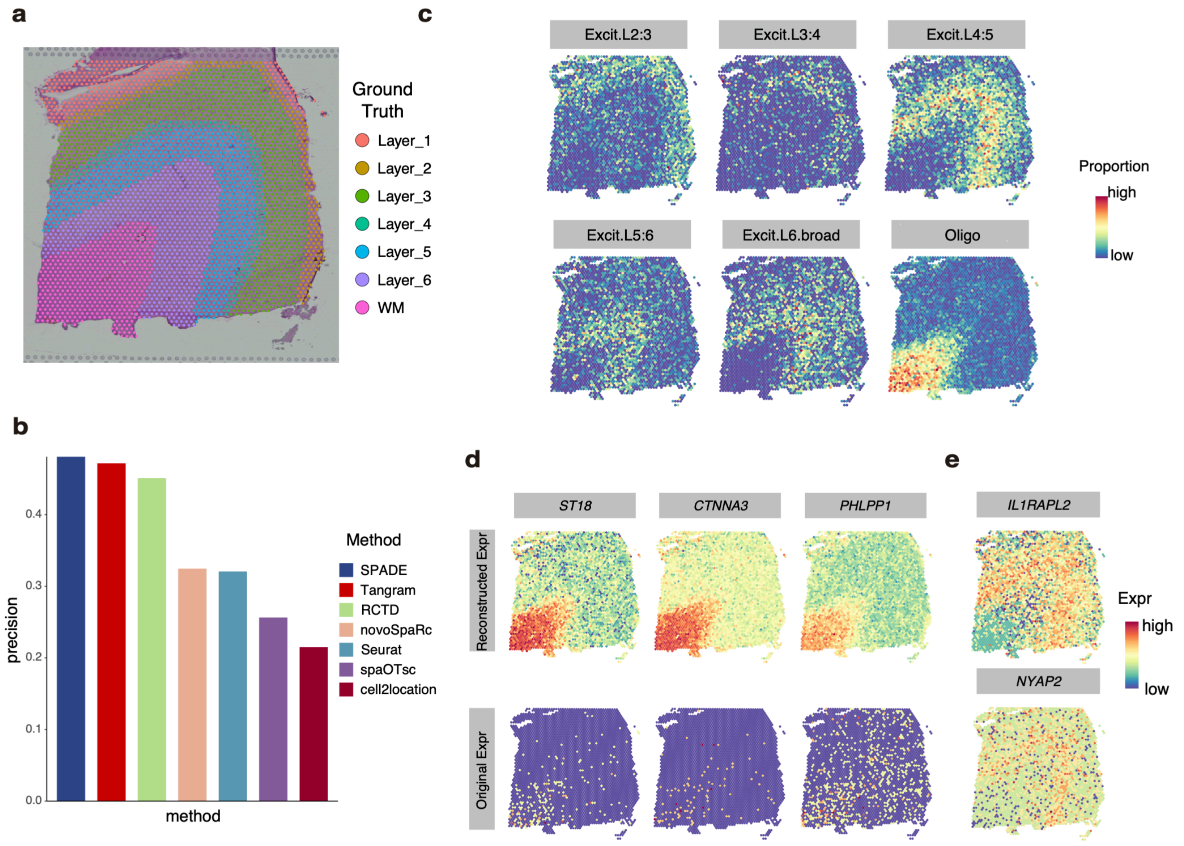

Supplementary Fig. 7. The alignment of SPADE in the human DLPFC dataset with known structures. (**a**) The ground truth of DLPFC structure, which includes six distinct layers and the white matter region. (**b**) The precision of cell type alignment by SPADE and other methods. (**c**) The alignment obtained from SPADE. (**d**) The gene expression enhancement by SPADE. Top line: the SVG expression of reconstructed ST profile; bottom line: the SVG expression of original ST profile. (**e**) The predicted expression of two SVGs that were not detected in the original dataset.

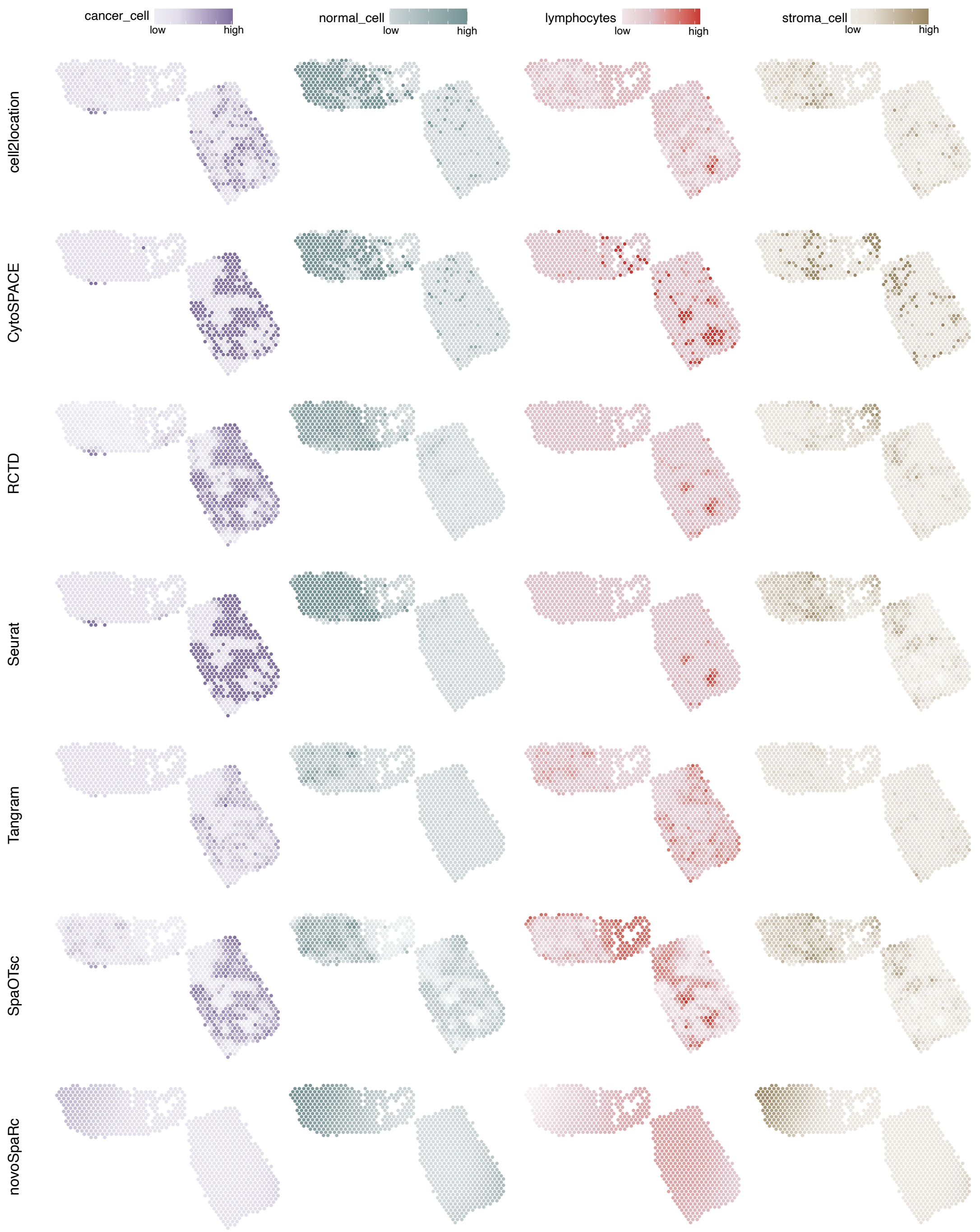

Supplementary Fig. 8. The mapping of major cell types to spatial in the TNBC dataset via seven methods.

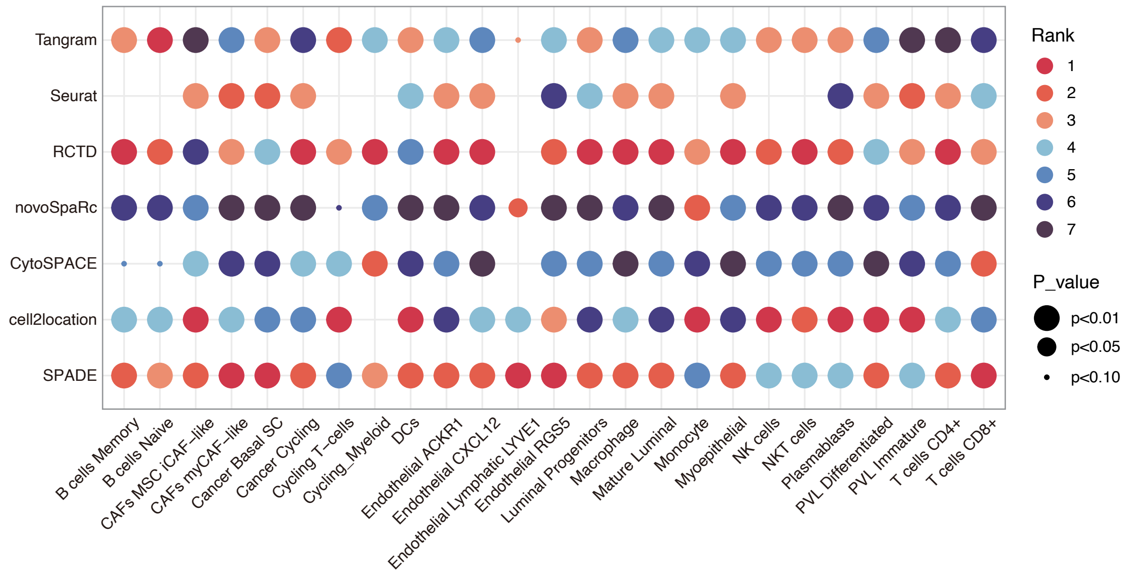

Supplementary Fig. 9. Benchmark using Pearson's correlation on TNBC datasets. Significance (point size) and rank (colors) of correlations between inferred cell-type proportions and corresponding cell-type-specific marker genes across spatial locations for each algorithm. *p*-values calculated through a two-sided *t*-test for the significance of Pearson's correlation.

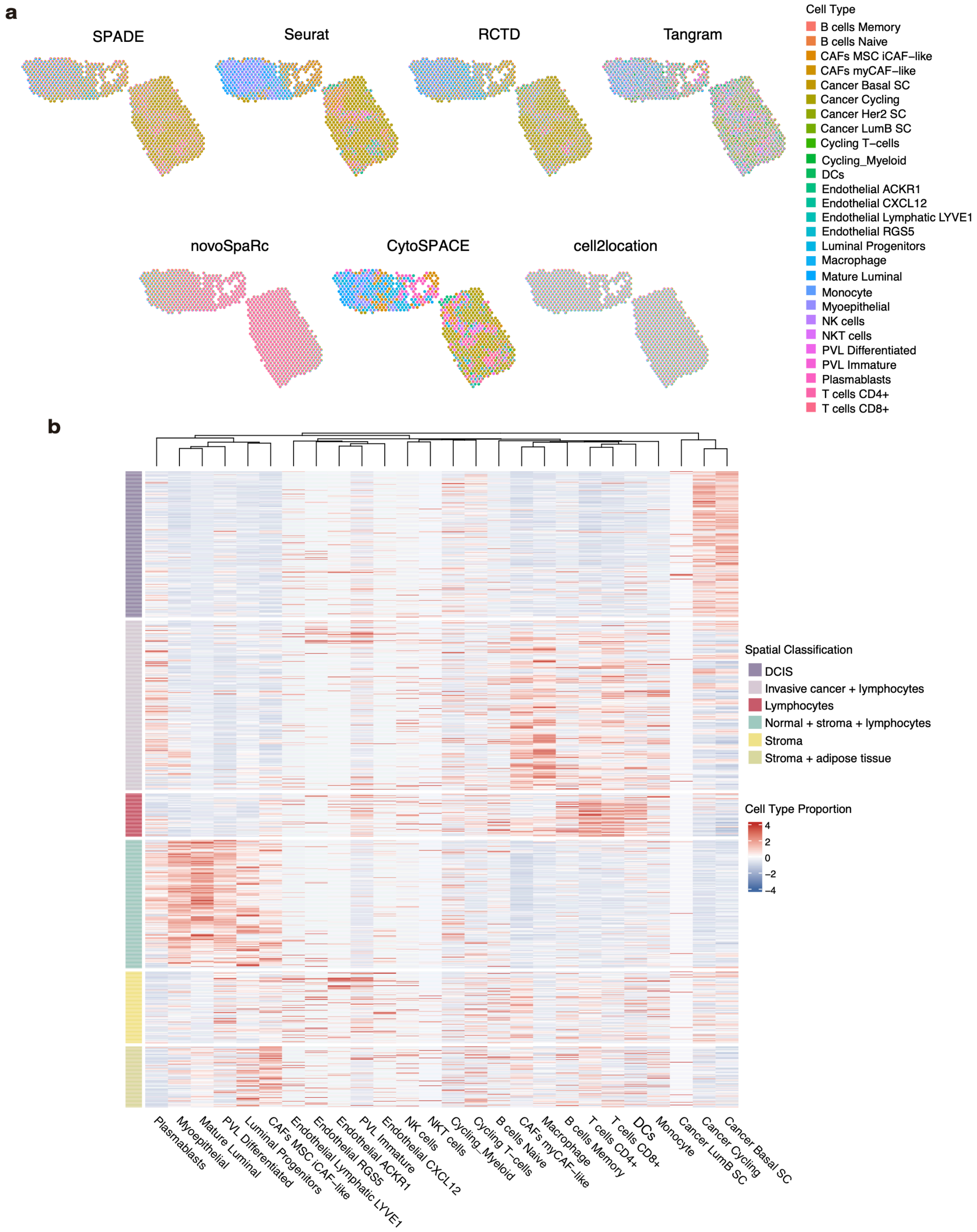

Supplementary Fig. 10. The mapping of scRNA-seq data to spatial in the TNBC dataset. (**a**) The cell type proportion of each spot estimated by six methods. (**b**) The cell type proportion of each spot estimated by SPADE. The color bars left of the heatmap indicate the spatial classification of each spot.

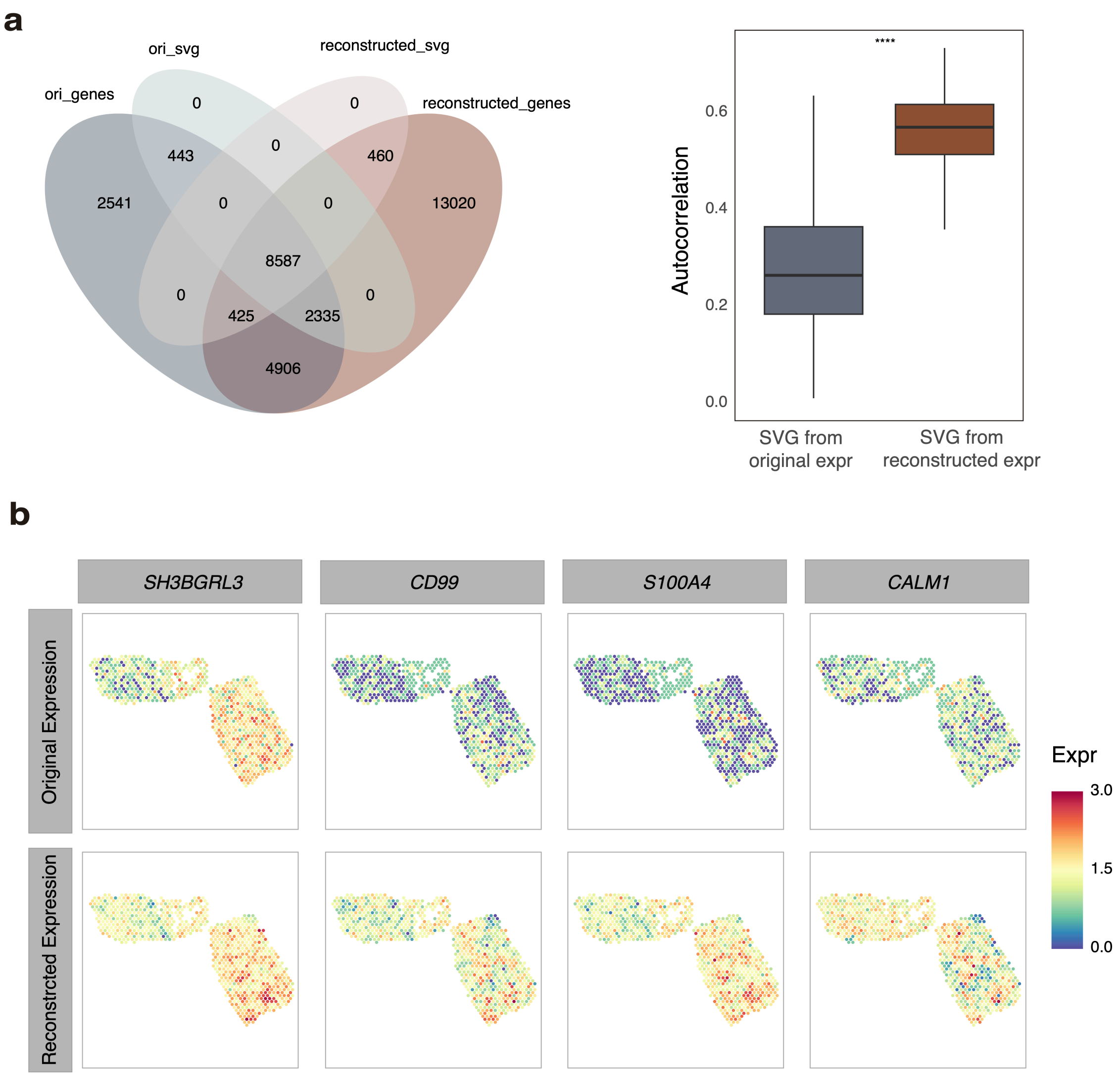

Supplementary Fig. 11. The performance of gene imputation in TNBC dataset. (**a**) Venn diagram of the detected genes and SVGs between original and SPADE-reconstructed spatial transcriptomics data (left) and comparisons of spatial autocorrelation of SVGs between the original and SPADE-reconstructed spatial transcriptomic data (right). (**b**) The gene expression enhancement of SPADE. The first line represents gene expression from original data, and the second line represents gene expression after reconstruction of SPADE.

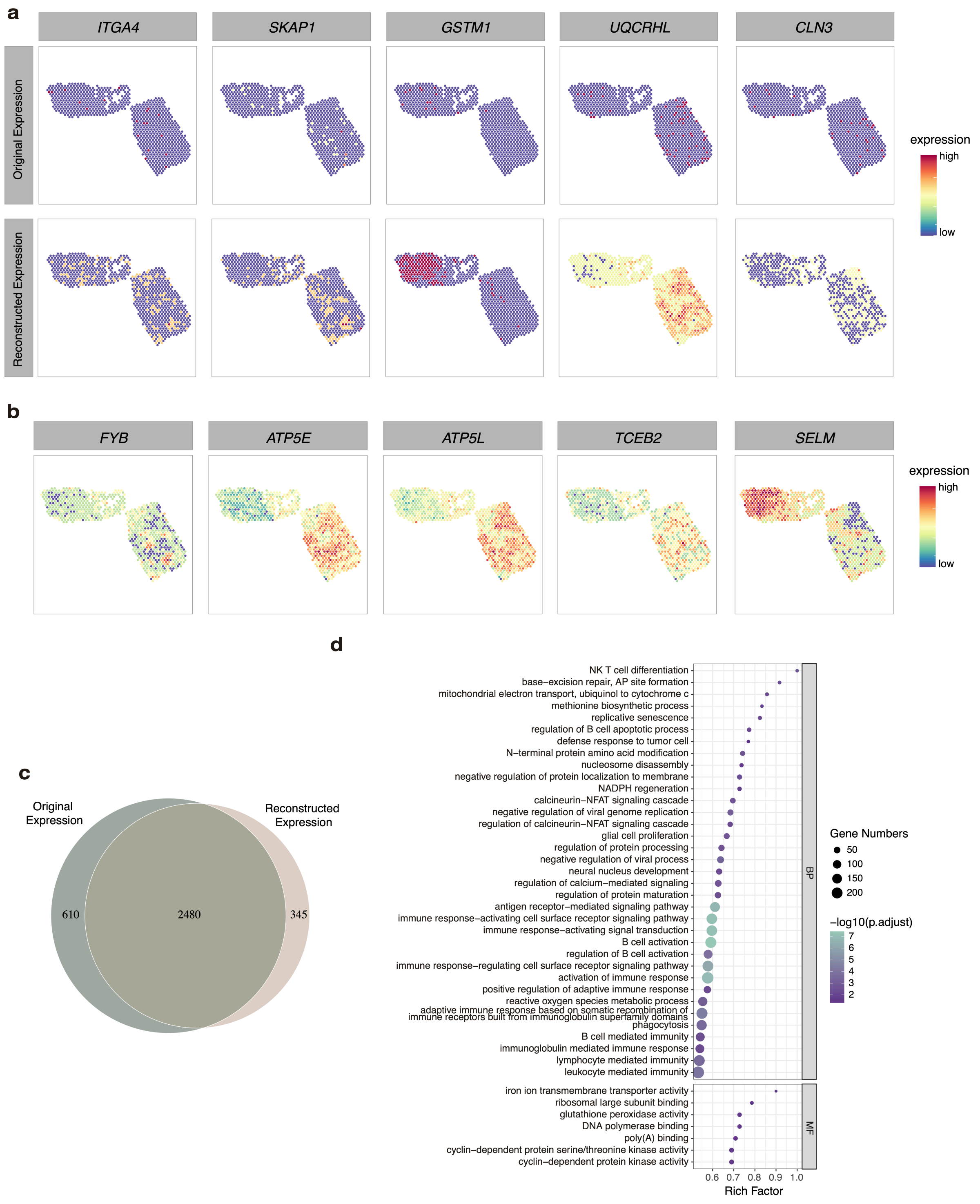

Supplementary Fig. 12. The reconstructed gene expression profile of the TNBC dataset. (**a**) The gene expression enhancement by SPADE. Top line: the SVG expression of reconstructed ST profile; bottom line: the SVG expression of original ST profile. (**b**) The SVG expression predicted by SPADE. These genes were not sequenced in the original dataset. (**c**) The Venn graph reveals the number of significant pathways that enriched by SVGs from original expression and reconstructed expression. (**d**) The enriched pathways by SVGs from SPADE reconstructed gene expression.

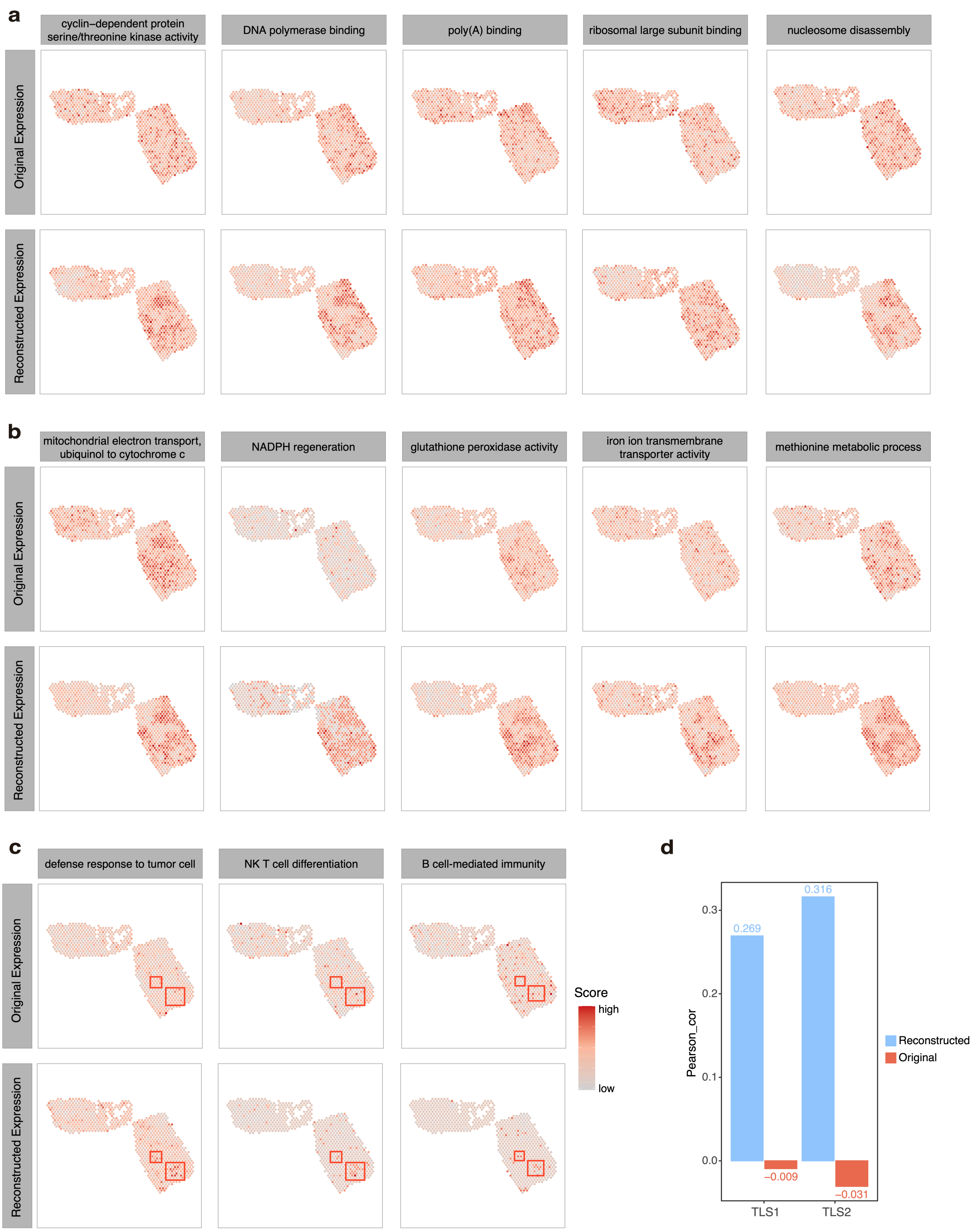

Supplementary Fig. 13. Scores of pathways uniquely enriched by SVGs from reconstructed profiles. The scores of pathways correlated with (**a**) tumor proliferation, (**b**) metabolism reprogramming, and (**c**) TME in the original and reconstructed ST profiles, red boxed represent TLS areas. (**d**) Pearson's correlation between B cell-mediated immunity pathway expression and TLS scores in the original and reconstructed ST profiles. Numbers above or below the bars indicate Pearson's correlation coefficients.

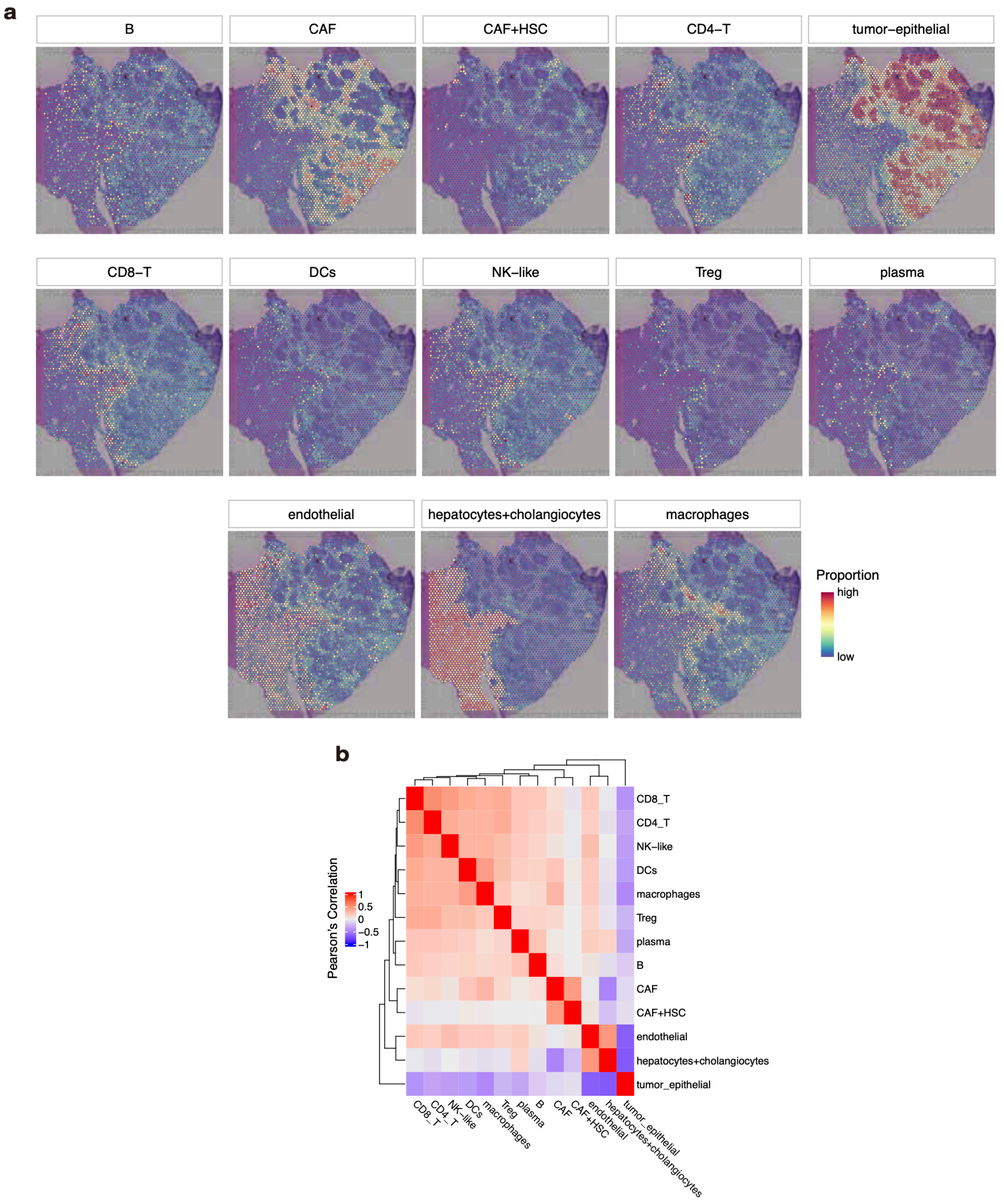

Supplementary Fig. 14. The spatial characteristic of the CRCLM dataset. (**a**) The cell type distribution estimated by SPADE. (**b**) Colocalization of cell types estimated by SPADE. The Pearson's correlations between cell type proportions are shown in the heatmap.

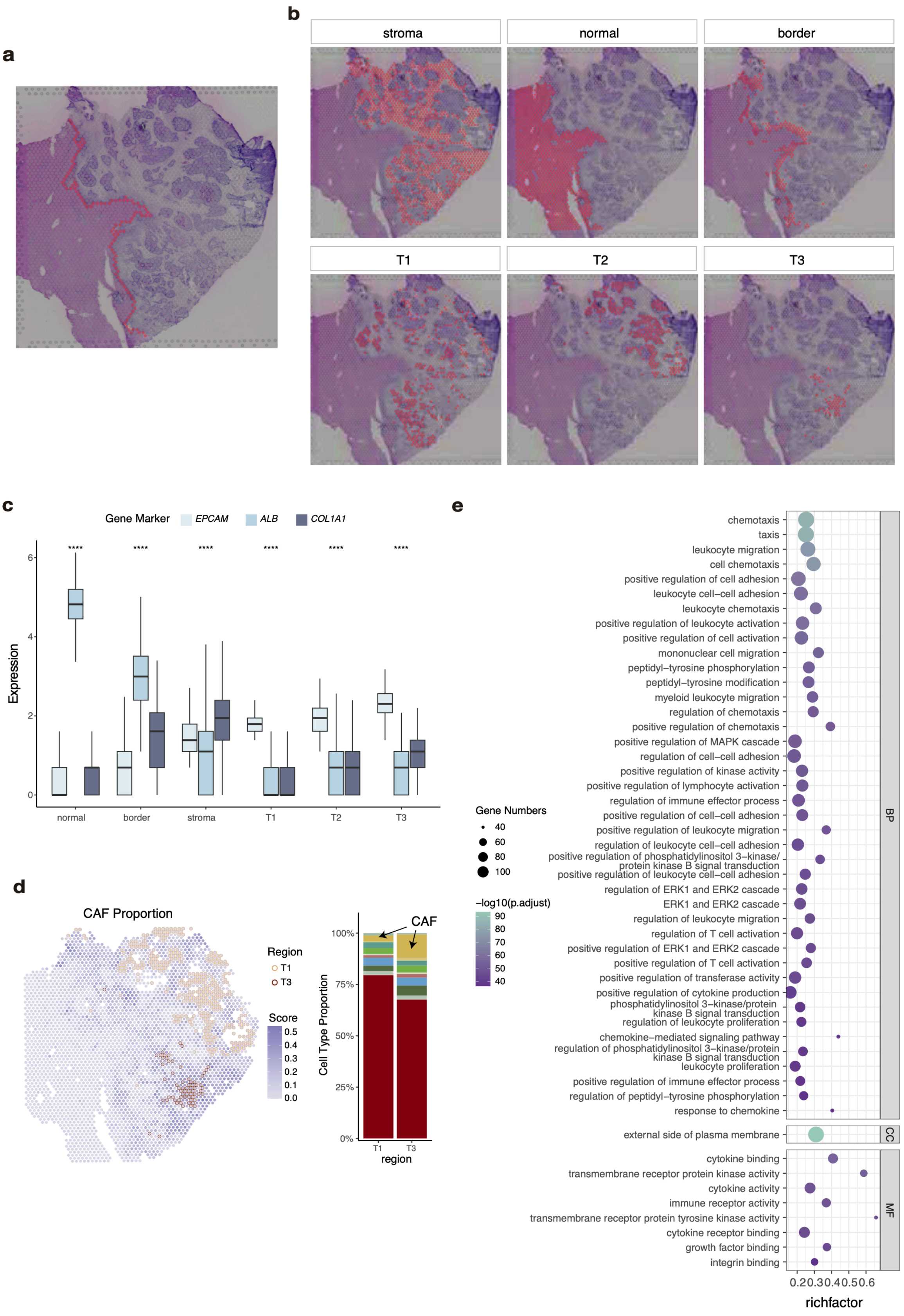

Supplementary Fig. 15. The analysis of the border region in CRCLM dataset. (**a**) The refined tumor-normal boundary based on histological features. (**b**) The six regions clustered in

the reconstructed ST profile. (**c**) The expression of three canonical marker genes in the six regions. One way analysis of variance (ANOVA) is performed to evaluate the significance of expression difference. (**d**) CAF proportion of each spot calculated by SPADE (left) and cell type composition in region T1 and T3 (right). (**e**) The GO enrichment analysis of the ligand and receptor genes significantly enriched in border region.

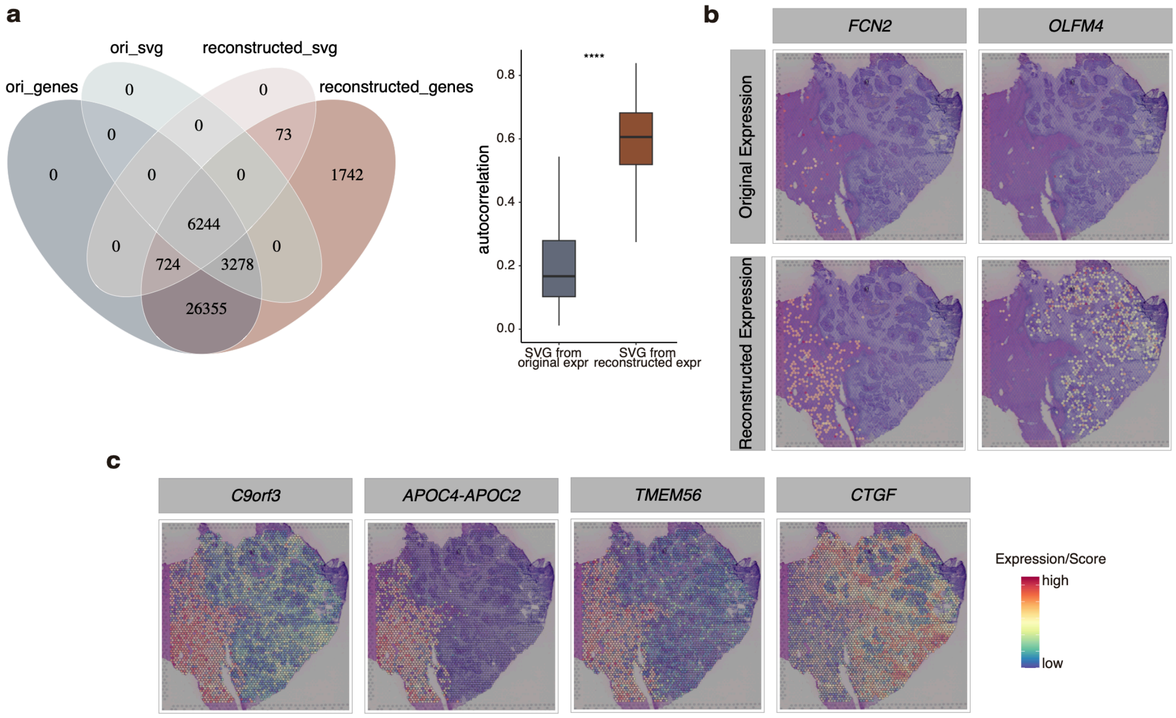

Supplementary Fig. 16. Comparison between original and reconstructed profiles. (**a**) Venn diagram of the detected genes and SVGs between original and SPADE-reconstructed spatial transcriptomics data (left) and comparisons of spatial autocorrelation of SVGs between the original and SPADE-reconstructed spatial transcriptomic data (right). **(b)** Spatial expression of SVGs newly identified by SPADE. The first line represents gene expression from original data, and the second line represents gene expression after reconstruction of SPADE. **(c)** Spatial expression of new SVGs imputed by SPADE.

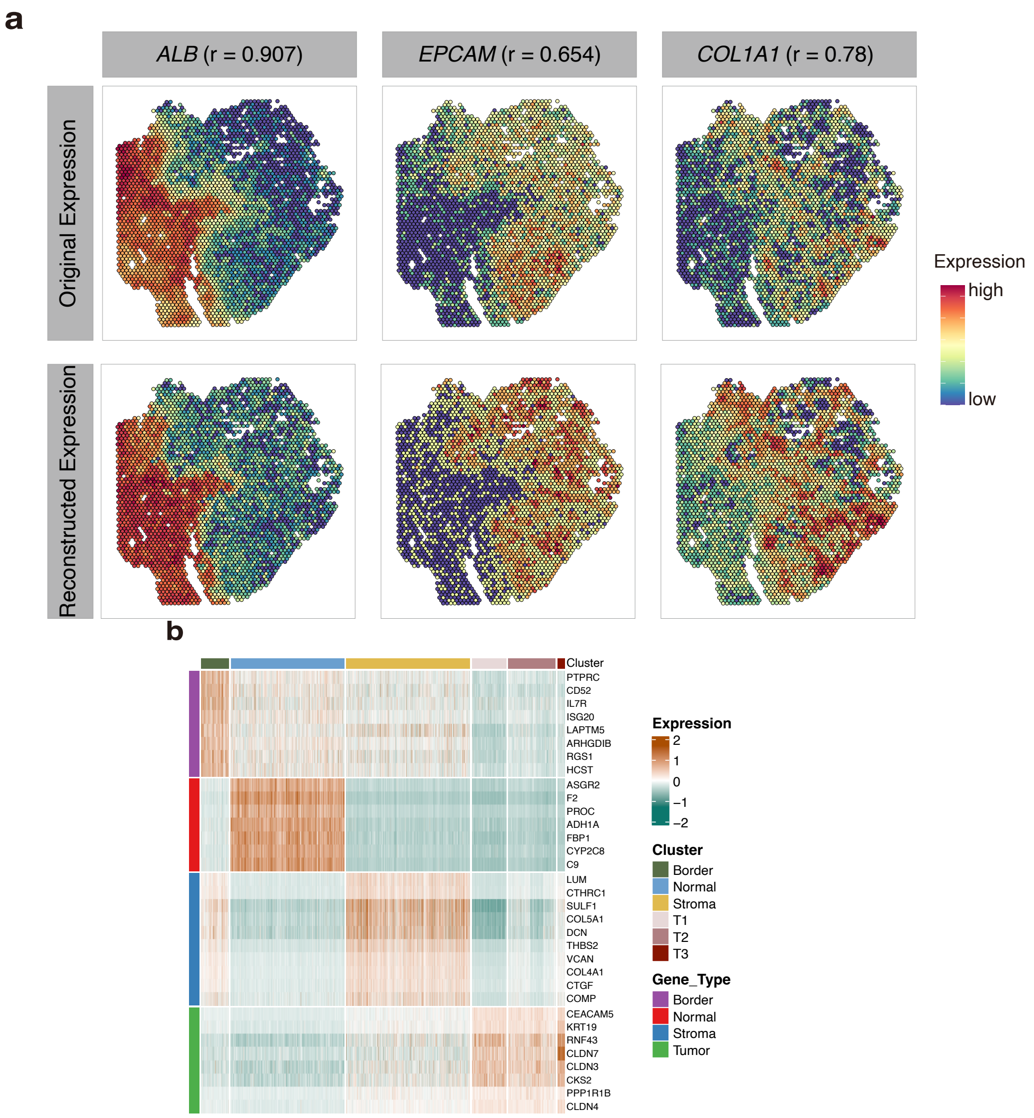

Supplementary Fig. 17. The gene expression analysis of four regions in CRCLM dataset. **(a)** Spatial expression patterns of three marker genes in original (top row) and reconstructed data (bottom row). Pearson's correlation coefficient between original and reconstructed spatial distribution for each gene is annotated on the gene name. (**b**) The top marker gene expression of six clusters.

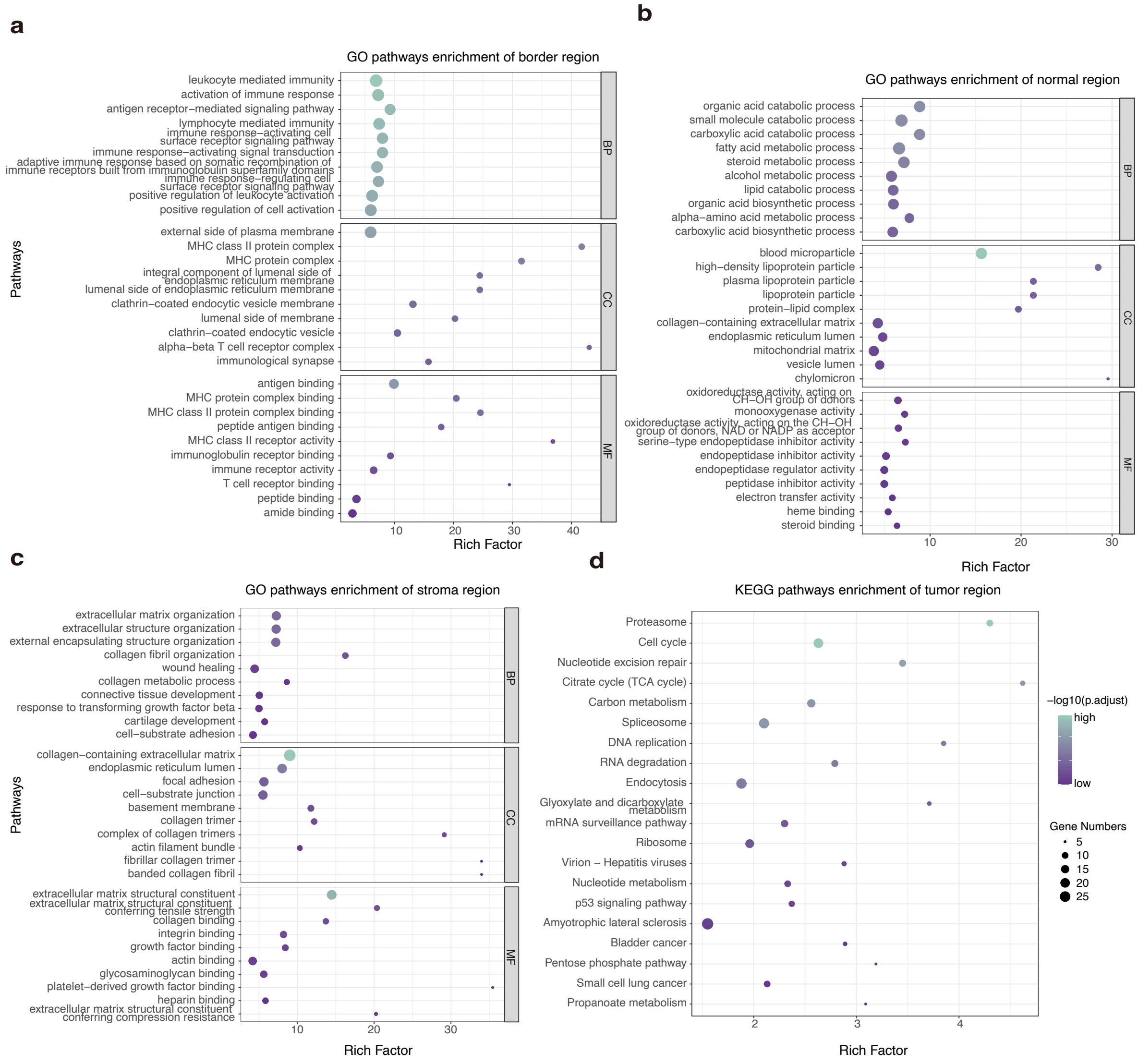

Supplementary Fig 18. The GO/KEGG enrichment analysis of four regions. Pathways significantly enriched in order region(**a**), normal region (**b**), stroma region (**c**), and tumor region (**d**).

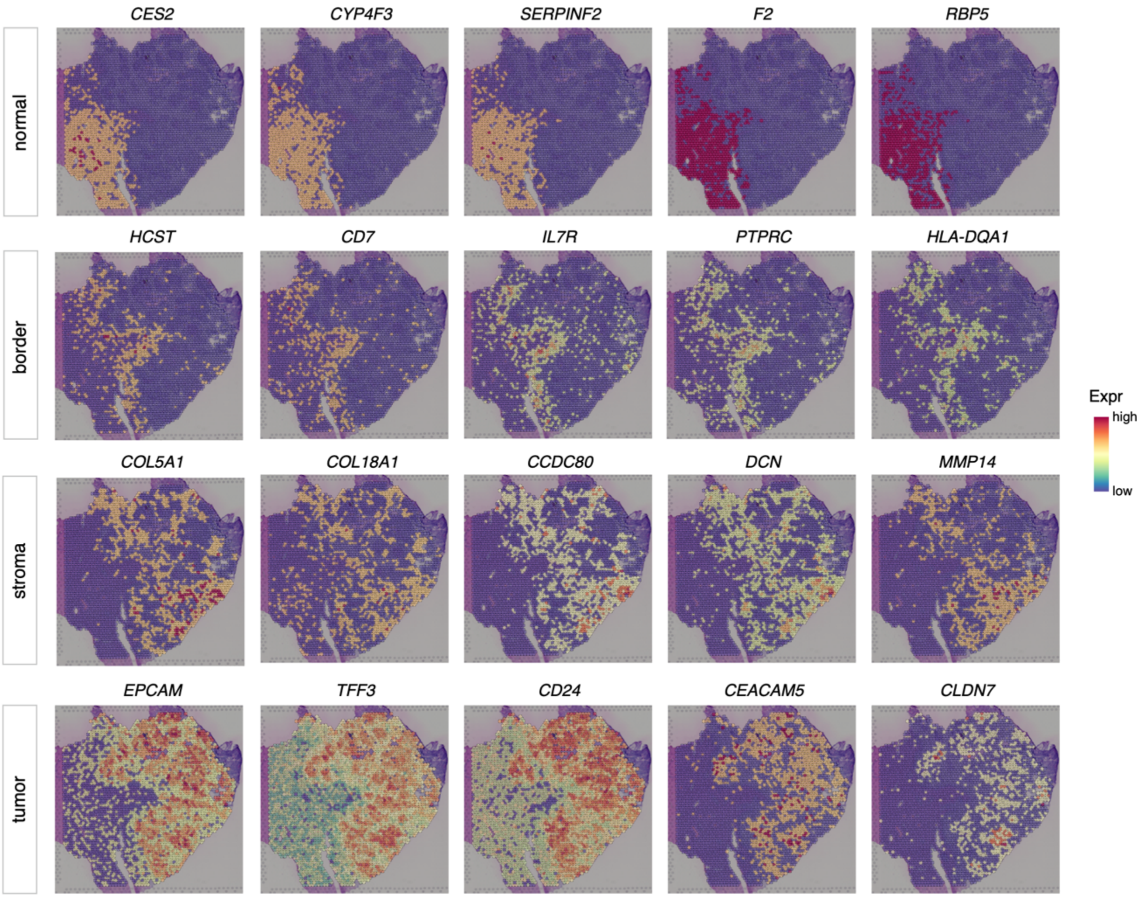

Supplementary Fig 19. The marker gene expression of each region. These genes exhibit highly region-specific expression patterns and matched functions.

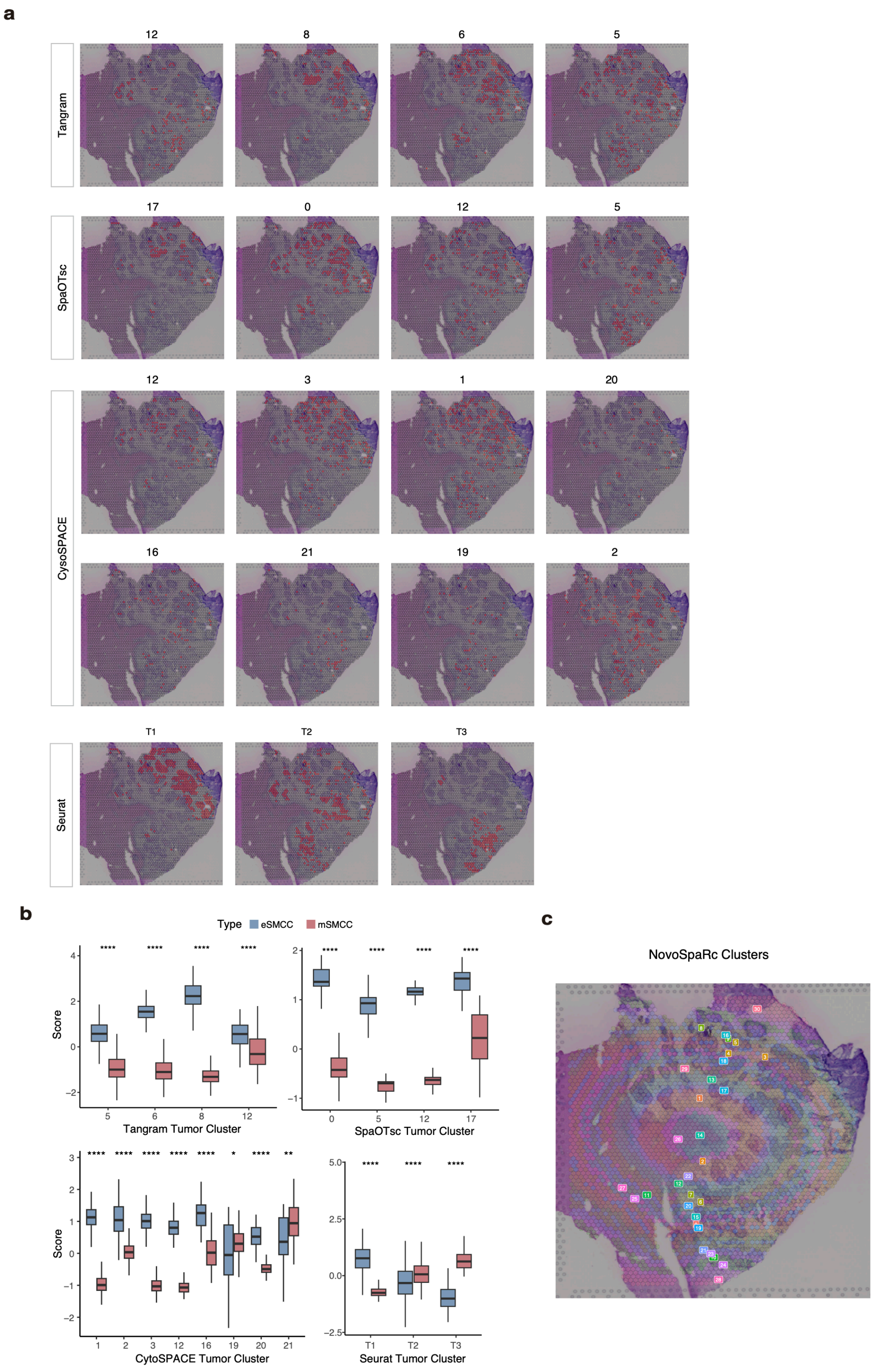

Supplementary Fig 20. The cluster results of five methods and SMCC subtype scores. (**a**) The tumor spatial domains calculated by the gene expression obtained from original ST or four methods. (**b**) eSMCC and mSMCC subtype scores across tumor clusters. (**c**) The spatial domains reported by NovoSpaRc.

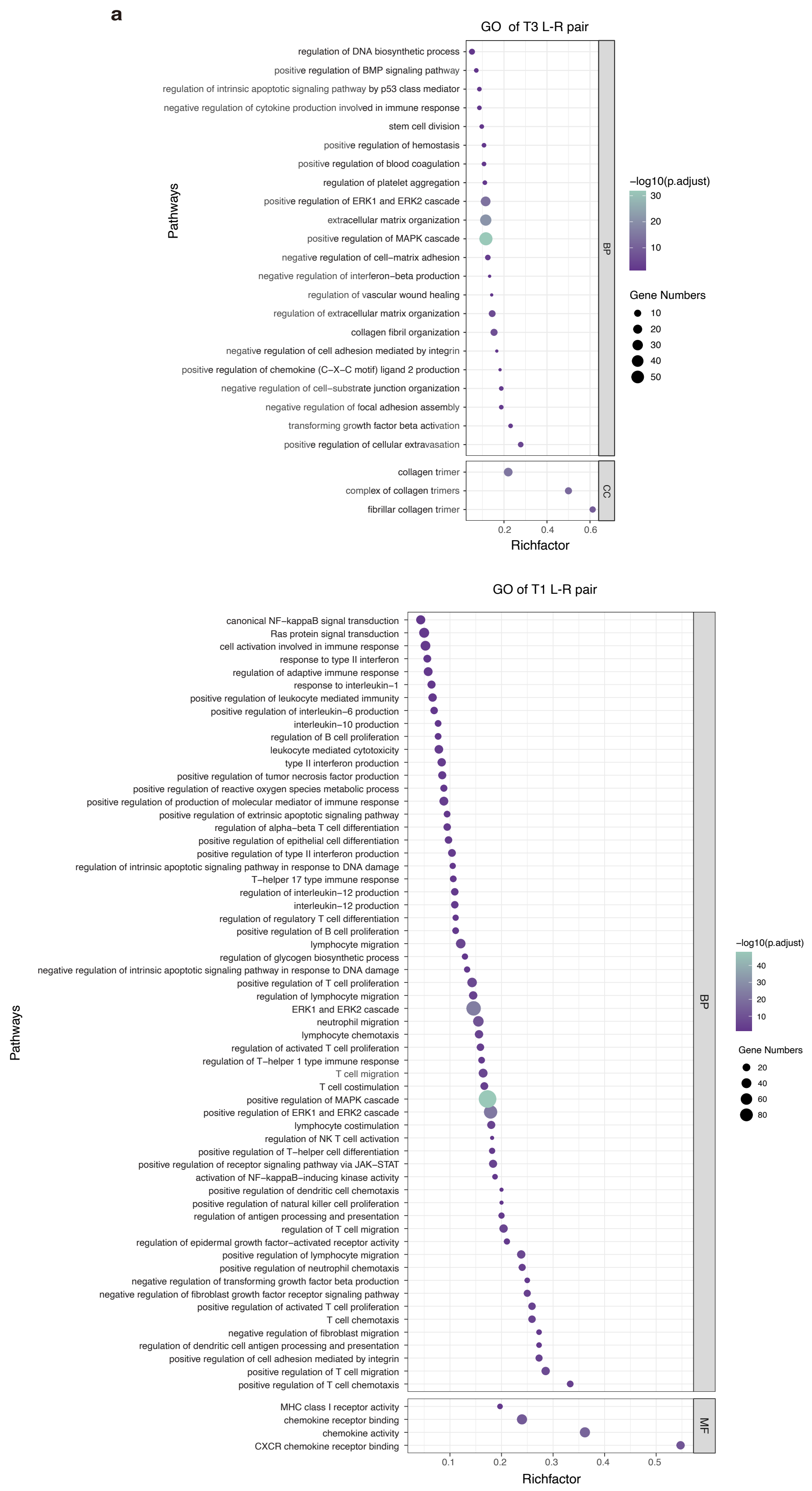

Supplementary Fig 21. The GO enrichment of ligand and receptor genes that were active in T1 (a) and T3 (b), respectively.

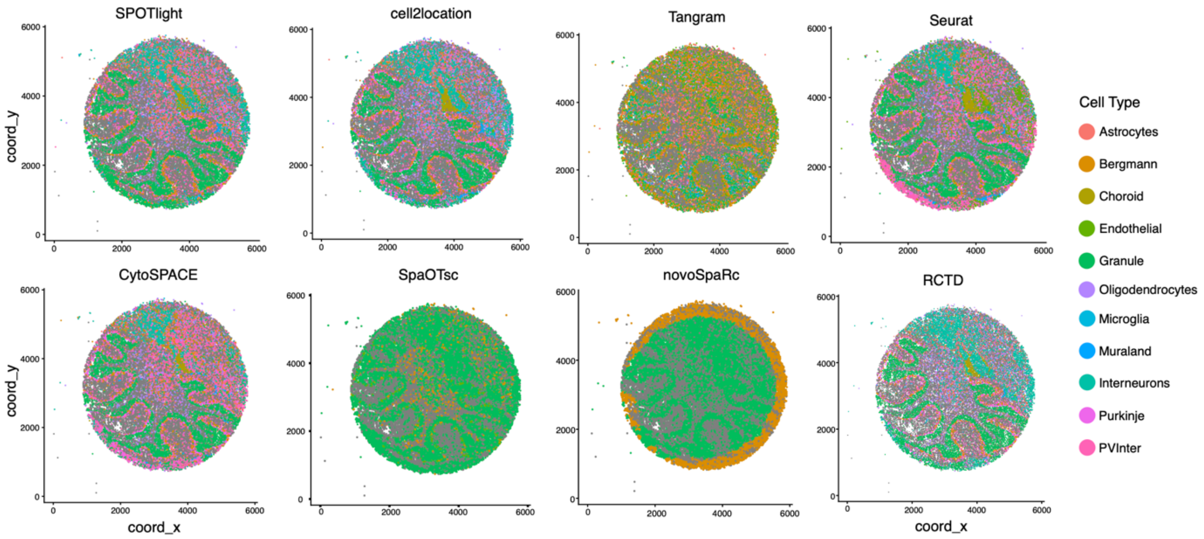

Supplementary Fig 22. The spot type annotation from eight methods. SPOTlight, cell2location, Seurat, CytoSPACE, and RCTD can largely reveal the structure.

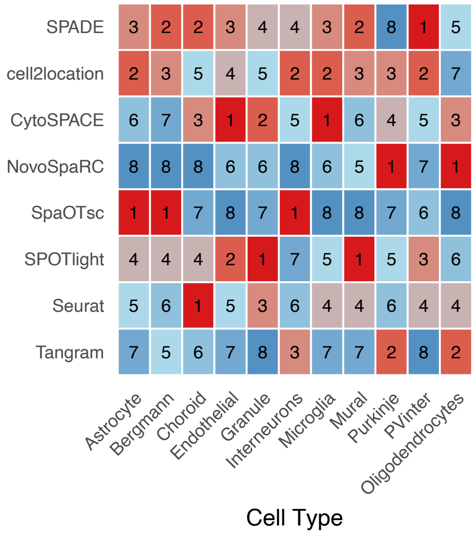

Supplementary Fig 23. The rank of correlation coefficient between gene module score and estimated cell type proportion.

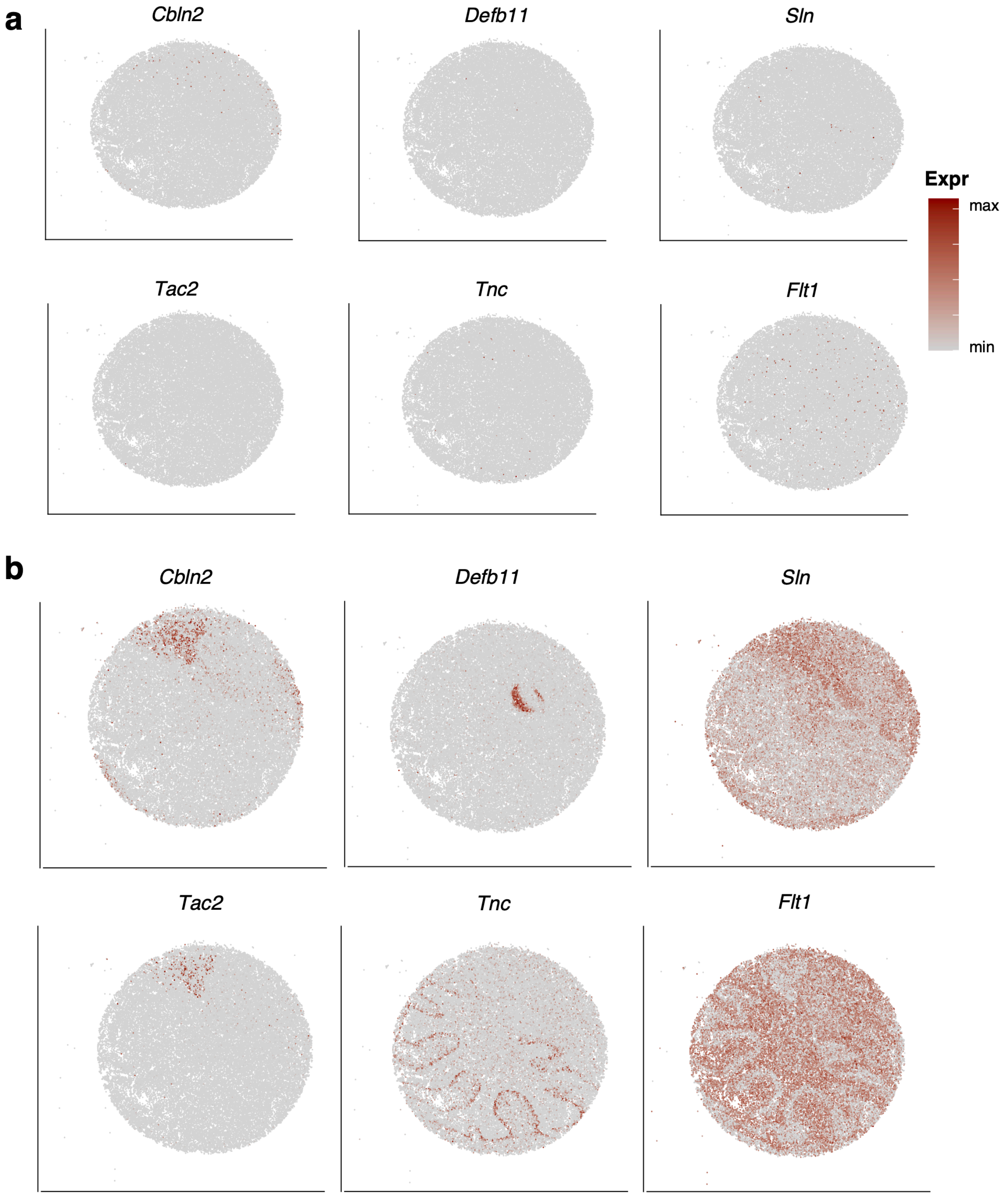

Supplementary Fig 24. Gene enhancement of SPADE in mouse cerebellum dataset. The original gene expression pattern (**a**) and the reconstructed gene expression patterns (**b**) of six genes.
